# Evidence for telemedicine heterogeneity in rheumatic and musculoskeletal diseases care: a scoping review

**DOI:** 10.1007/s10067-024-07052-w

**Published:** 2024-07-10

**Authors:** Khadija El Aoufy, Maria Ramona Melis, Camilla Elena Magi, Silvia Bellando-Randone, Matteo Tamburini, Giulia Bandini, Alberto Moggi-Pignone, Marco Matucci-Cerinic, Stefano Bambi, Laura Rasero

**Affiliations:** 1 Department of Health Science, University of Florence, Viale Largo Brambilla, 3 - 50134 Florence, Italy; 2 Department of Experimental and Clinical Medicine, University of Florence, Viale Largo Brambilla, 3 - 50134 Florence, Italy; 3 Department of Geriatric Medicine, Division of Rheumatology, Careggi University Hospital, Florence, Italy; 4 University of Florence, Viale Largo Brambilla, 3 - 50134 Florence, Italy; 5 Unit of Immunology, Rheumatology, Allergy and Rare Diseases (UnIRAR), IRCCS San Raffaele Hospital, Milan, Italy; 6 Università Vita Salute San Raffaele, Milan, Italy

**Keywords:** Digital health, Rheumatic and musculoskeletal disease, Telehealth, Telemedicine, Telenursing, Tele-rheumatology

## Abstract

Telemedicine and digital health represent alternative approaches for clinical practice; indeed, its potential in healthcare services for prevention, diagnosis, treatment, rehabilitation, and disease monitoring is widely acknowledged. These are all crucial issues to consider when dealing with chronic Rheumatic and Musculoskeletal Diseases (RMDs). The aim was to determine the current state of telemedicine in the field of rheumatology, considering the tools and devices in use as well as the Patient Reported Outcomes. A scoping review was performed following the PRISMA-ScR, retrieving articles through five databases from 1990 to 2022. Inclusion criteria were as follows: (I) adult patients with RMDs, (II) original research papers in the English language with available abstracts, and (III) telehealth and telemedicine are provided as healthcare services. Within the 62 included studies, multiple tools of telemedicine were used: 21/62 websites/online platforms, 18/62 mobile applications, 16/62 telephone contacts, 5/62 video-consultations, and 1/62 wearable devices. Outcomes were classified based on the economic, clinical, and humanistic framework. Clinical outcomes assessed through digital tools were pain, disease activity, and serum uric acid levels. Humanistic outcomes have been grouped according to four categories (e.g., mental and physical function, health management, and health perception). The heterogeneity of digital tools in the field of rheumatology highlights the challenge of implementing reliable research into clinical practice. Effective telerehabilitation models have been presented, and the use of a tight control strategy has also been mentioned. Future research should focus on establishing studies on other RMDs as well as summarizing and formulating clinical guidelines for RMDs.

**Table:** 

**Key Points**
• *Evidence for the usefulness of telemedicine and digital health for managing and monitoring rheumatic and musculoskeletal diseases is progressively increasing.* • *Several digital tools effectively measure clinical and humanistic and patient reported outcomes in rheumatic and musculoskeletal diseases.* • *Integrating diverse digital tools in rheumatology is challenging yet promising.* • *Future research should focus on developing standardized recommendations for practical use of telemedicine in daily practice.*

**Supplementary Information:**

The online version contains supplementary material available at 10.1007/s10067-024-07052-w.

## Introduction

About 1.71 billion of the global population is affected by rheumatic musculoskeletal diseases (RMDs), leading to disability, mortality, and morbidity [1, 2]. Almost one-third of the general population experience a problem linked to RMDs, with a prevalence that ranges from 9.8% to 33.2% [3]. The WHO reports that RMDs alone account for half of all chronic diseases, especially in older adults, and are the primary cause of pain and disability in Europe [4]. In Italy, 27% out of the 60 million adults more than 5 million experience chronic pain and is diagnosed with a RMD [5]. In the last decades, epidemiological data about RMDs has shown an increase due to population aging, higher survival rates, and the spread of early diagnosis. Consequently, an increasing pressure is generated on healthcare services, delaying in some cases the implementation of valid clinical guidelines [6–9]. In the majority of RMDs, chronicity is a key aspect, requiring intensive patients’ monitoring and regular follow-ups, that sometimes can be effectively delivered also through telehealth tools with successful results in terms of user’s outcomes (i.e., self-care, engagement, satisfaction) [10–12].

The active involvement of patients in the collection of data about their health outcomes through the Patients Reported Outcome Measures (PROMs) is a significant option within busy health services and from the perspective of patient empowerment [3]. In addition, periods of disease remission favored by therapeutic innovation/biological therapy allow for patients remote monitoring with specific characteristics of clinical stability (i.e., low disease activity or disease remission) and established diagnosis in some settings, like the outpatient clinics [8, 9, 12].

The advancement of telemedicine (TM) in rheumatology facilitates the provision of services such as prevention, diagnosis, treatment, rehabilitation, and follow-up, lessening the burden of the disease from the patients and care-givers perspective, yet some aspects still remain to be better defined [11]. The term “telehealth” refers to a wider range of non-physician services, including TM, telenursing, telepharmacy, and linguistic interpretation, as well as analog and audio–video out-of-office visits as alternatives to in-person healthcare. These terms can be used interchangeably with integrated remote care modalities, including mobile health and E-health platforms [13]. Specifically, the term e-Health has been used since 1990s to refer to the use of information and communication technology to support, improve, or provide access to knowledge and services in healthcare settings [14]. However, nowadays, it has come to refer to all services, goods, procedures, and infrastructures related to digitization in the healthcare industry [15].

Tele-rheumatology originated prior to the COVID-19 pandemic, yet it has played a crucial role with widespread utilization specifically during the pandemic, preventing the discontinuation of clinical care and helping to avoid patients’ isolation [15, 16].

During lockdown periods, authors from Asia, Europe, and the USA reported that the amount of remote and telematic healthcare provided to RMDs patients increased from 50 to 150% [8, 17–20]. According to Metha et al., 98% of rheumatologists modified their clinical practices during the pandemic, and 82% of them continue to use TM to guarantee continuity of care [21]. During that time, telemedicine also prevented contact between healthcare professionals (HCPs) and patients, which decreased the risk of cross-infection in hospitals and contagion anxiety among the population [22].

At the best of our knowledge, with an exponential increase in published research on telemedicine, there is a great study heterogeneity in terms of methods and digital tools used, making implications and recommendations for clinical practice challenging to produce. Thus, the aim of the present scoping review is to identify the state of the art of the role of telemedicine in the field of rheumatology, with reference to the device used and PROs included by the authors.

## Materials and methods

This scoping review was conducted following approaches outlined by Arksey and O’Malley and Levac et al., which provide a clear and rapid overview of relevant information regarding the telemedicine’s role in rheumatology [22, 23]. The scoping review protocol was developed following the Preferred Reporting Items for Systematic Reviews and Meta-Analyses Extension for Scoping Reviews (PRISMA-ScR) [24]. Ethical approval was not sought as the data were publicly available.

The team conducted this scoping review through the following phases: (I) identifying the research question; (II) identifying relevant studies; (III) selecting the studies; (IV) extracting and charting the data; and (V) collating, summarizing, and reporting the results.

### Phase I: identifying the research question

A research question was identified in accordance with the PEOS (Population, Exposure, Outcomes, and Study type) methodology (Table 1). RMDs patients were selected due to the prevalence of the disease and its high rate of complications. The exposure chosen included telemedicine, telenursing, and telehealth intervention, due to their growing utilization in healthcare and their widespread availability to persons nowadays. Telemedicine was defined as the utilization of remote communication technology aimed at treating, advising, screening, or reminding patients about their monitoring and treatment (e.g., text messaging, telephone communication, videoconferencing, and the use of mobile applications). Indeed, TM, in the present scoping review, refers to the provision of healthcare in situations where the health professional and the patient are not in the same physical location; then communication between the patients and the health professionals is activated, and data and information are moved.

**Table 1:** PEOS of the scoping review

Types of participants (P)	Adult patients affected by RMDs
Type of exposure (E)	Telemedicine, telenursing and telehealth intervention
Type of outcomes (O)	Patient-reported outcome, patient’s perception, patient satisfaction, quality of life, disease activity, self-care, self-management, self-efficacy
Type of studies (S)	Any kind of (hospital and primary care)

The team hypothesized that employing telemedicine could enhance self-care among patients with RMDs and result in better economic, clinical, and humanistic outcomes (ECHO) [25].

Patients with RMDs were defined as adults (≥ 18 years old) affected by RMDs according to the American College of Rheumatology/European Alliance of Associations of Rheumatology (ACR/EULAR).

As for the outcome, disease activity and ECHO were included in the review. Disease activity likely refers to the level of severity or progression of the RMDs being studied. As for ECHO, it stands for economic, clinical, and humanistic outcomes, indicating a comprehensive approach to assessing the impact of the intervention. Economic outcomes were not searched within the field of the present scoping review, yet identified if present in the included papers, based on the definitions of direct and indirect cost, as measures of medical resource utilization such as hospitalization and medication costs, or evaluations of reduced productivity and lost working days. Clinical outcomes were defined as medical events that occurred because of healthcare services, including rate of readmission, emergency department visit, mortality, adverse drug events (including all-cause and serious adverse events), blood, and physiological parameters. Humanistic outcomes were defined as the consequences of disease or treatment on patient functional status or quality of life, including physical and social function, general health and well-being, and life satisfaction, including the relationship with the healthcare system (i.e., satisfaction with care, quality care).

### Phase II: identifying relevant studies

In order to answer our research question and to identify the existing literature, the electronic databases MEDLINE, CINAHL (Cumulative Index to Nursing and Allied Health Literature), EMBASE (Excerpta Medica Database), APA (American Psychological Association), PsycINFO, and SCOPUS were investigated, using the following keywords to develop the search strategy for this scoping review: “rheumatic diseases,” “telemedicine,” “telenursing,” “telehealth,” and “patient reported outcome.” The search strategy process was supervised and guided by the biomedical librarian.

After reading several documents, a search strategy combining Medical Subject Headings (MeSH) terms and free words was developed (Table 2). The different strings were divided into 3 dimensions: that relating to rheumatic diseases, that relating to telemedicine, and that relating to various patient outcomes. Each of these dimensions is joined by the Boolean operator “AND.” Keywords were chosen either as the terms of the Thesaurus (MeSH for MEDLINE, Headings for CINAHL, and EMTree for EMBASE) or as common words combined through the Boolean operators “OR,” “AND,” and “NOT.” Published records between January 1, 1990, and December 6, 2022, were downloaded for the screening process.

**Table 2:** Search strategy used for PubMed (MEDLINE) database

PUBMED
((rheumatic diseases[MeSH Terms]) OR (rheumatic disease[Title/Abstract]) OR (rheumatology[MeSH Terms]) OR (rheumatolog*[Title/Abstract]) OR (rheumatic condition[Title/Abstract]) OR (rheumatic disorder[Title/Abstract]) OR (arthritis rheumatoid[MeSH Terms]) OR (rheumatoid arthritis[Title/Abstract]) OR (arthritis, juvenile[MeSH Terms]) OR (arthritis juvenile[Title/Abstract]) OR (juvenile idiopathic arthritis [Title/Abstract]) OR (psoriatic arthritis[Title/Abstract]) OR (reactive arthritis[Title/Abstract]) OR (osteoarthritis[MeSH Terms]) OR (osteoarthritis[Title/Abstract]) OR (spondyloarthropathies [MeSH Terms]) OR (spondyloarthropathies[Title/Abstract]) OR (spondylitis ankylosing[MeSH Terms]) OR (spondylitis ankylosing[Title/Abstract]) OR (polymyalgia rheumatica[MeSH Terms]) OR (polymyalgia rheumatica[Title/Abstract]) OR (mixed connective tissue disease[MeSH Terms]) OR (mixed connective tissue disease[Title/Abstract]) OR (lupus erythematosus, cutaneous[MeSH Terms]) OR (lupus erythematosus cutaneous[Title/Abstract]) OR (Lupus erythematosus systemic [MeSH Terms]) OR (lupus[Title/Abstract]) OR (scleroderma, systemic[MeSH Terms]) OR (scleroderma, systemic[Title/Abstract]) OR (scleroderma[Title/Abstract]) OR (systemic sclerosis[Title/Abstract]) OR (sjogren's syndrome[MeSH Terms]) OR (sjogren's syndrome[Title/Abstract]) OR (vasculitis[MeSH Terms]) OR (vasculitis[Title/Abstract]) OR (myositis[MeSH Terms]) OR (myositis[Title/Abstract]) OR (fibromyalgia[MeSH Terms]) OR (fibromyalgia[Title/Abstract])) **AND** ((telemedicine[MeSH Terms]) OR (telemedicine[Title/Abstract]) OR (telenursing[MeSH Terms]) OR (telenursing[Title/Abstract]) OR (mobile health[Title/Abstract]) OR (health mobile[Title/Abstract]) OR (mhealth[Title/Abstract]) OR (telehealth[Title/Abstract]) OR (ehealth[Title/Abstract]) OR (digital medicine[Title/Abstract]) OR (remote consultation [Title/Abstract])) **AND** ((patient reported outcome measures[MeSH Terms]) OR (patient reported outcome measures[Title/Abstract]) OR (PROMs[Title/Abstract]) OR (PROMIS[Title/Abstract]) OR (PRO-PM[Title/Abstract]) OR (e-PROM*[Title/Abstract]) OR (patient-reported outcome*[Title/Abstract]) OR (patient reported outcome*[Title/Abstract]) OR (patient care management[MeSH Terms]) OR (patient care management[Title/Abstract]) OR (continuity of patient care[MeSH Terms]) OR (continuity of patient care[Title/Abstract]) OR (self management[MeSH Terms]) OR (self management[Title/Abstract]) OR (self efficacy[MeSH Terms]) OR (self efficacy[Title/Abstract]) OR (self care[MeSH Terms]) OR (self care[Title/Abstract]) OR (patient care[MeSH Terms]) OR (patient care[Title/Abstract]) OR (patient's perception[Title/Abstract]) OR (patient satisfaction[Title/Abstract]) OR (quality of life[Title/Abstract]) OR (QoL[Title/Abstract]) OR (disease activity[Title/Abstract]) OR (HRQoL[Title/Abstract]) OR (health-related quality of life[Title/Abstract]) OR (health related quality of life[Title/Abstract]) OR (Patient Compliance[MeSH Terms]) OR (Patient Compliance[Title/Abstract]) OR (disease management [Title/Abstract]))

Records published between January 1, 1990, and December 6, 2022, were included for screening, limited to English language articles due to resource constraints. Exclusions comprised letters, editorials, news articles, reviews, theses, and other non-original qualitative or quantitative research papers. Additionally, studies lacking information about patients with RMDs or where telemedicine intervention was not explicitly detailed were excluded. Studies involving physicians, advanced practice nurses, physician associates, registered nurses, or pharmacists as presenters were included in the search.

### Phase III: selecting the studies

The team that conducted the scoping review consisted of three nurses (KEA, MT, MRM) and a supervisor for the screening part with the role of resolving disagreements between the three reviewers (SB). The team members discussed and agreed on the relevance of the research question, examined whether the study design was appropriate, elaborated, and agreed on the research strategy. Retrieved literature was managed using EndNote™ (software version X9.3.3), which allowed the identification of duplicates to delete, according to literature title. After, titles and abstracts were screened independently by two reviewers (KEA and MRM, with the support of MT) according to the eligibility criteria. Then, retrieval of the full text was independently assessed in accordance with the inclusion/exclusion criteria. In case of disagreement, another reviewer (SB) was consulted. The study selection process is reported in the PRISMA flowchart.

### Phase IV: extracting and charting the data

Data from each eligible article were extracted and compiled using a standardized excel sheet that was designed to guide the extraction of information from the records in accordance with the aim of the study, consisting of the following: authors, year and country of publication, study aim, study design, population and setting, intervention, results, outcomes measured, and tools used to measure them (Tables 3 and 4). Moreover, outcomes present in the included studies were divided following the ECHO model for the clinical, economic, and humanistic outcomes [25, 26].

**Table 3:** Data extraction for quantitative studies

Author (year) Country	Study aim	Study design	Sample characteristics (mean age, % female, educational level)	Intervention and follow-up	Outcomes measured and tools	Results
Al Harashet al. (2021) USA [55]	To design the gout disease management program (GDMP) to improve outcomes, increase patient satisfaction, and decrease healthcare utilization	Experimental study	112 gout patients/mean age 60 years/13% females N/A	GDMP: monthly tailored telephone contact until serum uric acid (SUA) level = 6 mg/dl and pts flare free for 3 months (or 6 months for tophaceous gout), then every 6 months for 2 encounters, and finally, yearly if stable. No HPR was involved, only rheumatologists - 3 months–6 months–12 months if stable	1) SUA levels 2) Self-reported adherence 3) Patient satisfaction with GDMP (a 5-point Likert scale)	79 patients (70%) achieved the SUA goal of ≤ 6.0 mg/dl. Only 3 patients (2.6%) required hospitalization/ER visit or urgent care center due to gout flare, and 98% rated their encounter as a 5 on the 5-point Likert scale (patients satisfaction)
Alasfour M. and M. Almarwani (2022), Saudi Arabia [49]	To examine the effects and effectiveness of an Arabic smart app on adherence to home exercise programs (HEPs) on pain, physical function and lower-limb muscle strength among older women with KOA	RCT parallel study	40 KOA/20 pts/mean age of 54.40 ± 4.33 years Educational level IC –IG primary, *n* (%) IG *n* = 3 [15], CG *n* = 10 [50] Intermediate, *n* (%) IG *n* = 9 [45], CG *n* = 2 [10] High, *n* (%) IG *n* = 8 [40], CG *n* = 8 [40]	For both groups (app group and paper group) 6 weeks exercise program included: isometric quadriceps contraction, isotonic quadriceps contraction, isotonic hamstring contraction, isotonic quadriceps contraction with resistance band, straight leg raising, side-lying hip abduction, partial squats, dynamic stepping exercise, and sidestepping with a resistance band around the thighs or ankles. The app group received home exercise programs (HEPs) as hand-outs/HPR involved only physiotherapist (PTs) - N/A	Adherence rate to smart app: self-reported adherence Pain reduction: Arabic NRS Physical function: the Arabic version of the reduced Western Ontario, McMaster Universities Osteoarthritis Index-Physical Function subscale Muscle strength: Five-Times Sit-To-Stand Test scores	At the end of week 6, the app group reported greater adherence rate (26.60%) to HEPs (*p* = .002) and significant reduction in pain (*p* = .015)
Allam et al. (2015), Switzerland [39]	To assess the effects of a Web-based intervention that included online social support features and gamification on physical activity, healthcare utilization, medication overuse, empowerment, and RA knowledge of RA patients	5-arms RCT	157 RA patients/115 pts intervention groups/mean age was 58.0 (SD 12.3) years/45.8% female Education level, *n* (%) Elementary school: IG *n* = 15 [13], CG *n* = 7 [18] Middle school: IG *n* = 15 [13], CG, n = 11 [28] High school: IG *n* = 83 [72], CG *n* = 19 [49] University: IG *n* = 11 [10], CG *n* = 2 [5]	4 different types of access to online social support and gamification features and 1 control group that had no access to the website. No HPR involved- online support - 2 months follow-up	Self-reported: physical activity, healthcare utilization, medication overuse; empowerment and RA knowledge	An important factor in raising patients’ levels of empowerment is online social support, which also contributes to lower healthcare and pharmaceutical overuse as well as greater empowerment. Gamification either by itself or in conjunction with social support enhanced exercise and self-efficacy and reduced use of medical services
Ammerlaan et al. (2014), Netherlands [56]	To test the feasibility of the online and face-to-face self-management program	Quantitative feasibility study	22 young adults with arthritis	12 young adults followed the online program and 10 followed the face-to-face program	Self-efficacy, self-management, user-acceptance, adherence	Physical activity increased over time for patients—social support + gaming (*P* = .02), decreased health care utilization for patients -social support (*P* = .01), and patients social support + gaming (*P* = .03). Patients who had access to either social support OR gaming gained more empowerment (*P* = .03; *P* = .05; respectively). Patients gaming experience used the website more often than the ones without gaming (*P* = .02; *P* = .02)
Baker et al. (2020), USA (40)	To determine whether the Boston Overcoming OA through Strength Training Telephone-Linked Communication (BOOST-TLC) program counselling intervention, improved adherence to a strength-training exercise program over 2 years	Single blind, parallel-arm RCT	104 patients with OA, 89 OA pts/45 TLC/mean age IG 61.1 ± 6.9, CG 63.4 ± 7.8% of female: CG *n* = 43 (82.7%); IG *n* = 42 (80.8%) Level of education was not specified	Group exercise class participation-then randomization to receiving motivational telephone calls through the BOOST-TLC program for 24 months or the control. Both control and intervention participants received a monthly automated phone message reminder to continue the program- Outcomes were evaluated at 6, 12, 18, and 24 months - 24 months follow-up	Exercise adherence was ascertained by a single self-report item scored 0–10, where 10 represented complete adherence	No changes of RA knowledge levels over time for any of the experimental conditions
Bennell et al. (2017), Australia [41]	To investigate whether simultaneous telephone coaching improves the clinical effectiveness of a physiotherapist-prescribed home-based physical activity program for KOA	RCT	168 adult [84 in each group]: age (years SD) PT + coaching group: 61.1 (6.9)—PT group: 63.4 (7.8) with knee pain on a NRS ≥ 4 (NRS; range 0–10) and knee OA (sex not specified) Level of education PT + coaching group PT group (*n*-%)—< 3 years of high school *n* = 6 (7%)/*n* = 12 (14%) ≥ 3 years of high school *n* = 33 (30%)/*n* = 30 (36%)- tertiary level *n* = 45 (54%)/*n* = 42 (50%)	Subjects randomly assigned to a physiotherapist (PT) + coaching group (*n* = 84) or PT-only (*n* = 84) group All participants received 5 × 30-min consultations with a PT over 6 months for education, home exercise, and physical activity advice. PT + coaching participants also received 6–12 telephone coaching sessions by clinicians trained in behavioral-change support for exercise and physical activity - 6 months follow-up 3	Primary outcomes were pain (NRS) and physical function (Western Ontario and McMaster Universities Osteoarthritis Index [WOMAC; score range 0–68]) at 6 months. Secondary outcomes were these same measures at 12 and 18 months, as well as physical activity, exercise adherence, other pain and function measures, and QoL	Both programs appeared to be feasible, especially in dealing with problems in daily life, and the participants indicated the time investment as “worthwhile.” In using the online program, no technical problems occurred. Participants found the program easy to use and liked the “look and feel” of the program
Berdal et al. (2018), Norway [57]	To evaluate patient-reported health effects of an add-on structured goal-planning and supportive telephone follow-up rehabilitation program compared with traditional rehabilitation programs in patients with RMDs	Multicentric RCT [6 centers]	389 RMDs pts/194 IG/ mean age (min/max) CG 57 [24, 89], IG 58 [23, 89]/sex F% IG 65.1%, CG 75.8% Education > 12 years, *n* (%): IG 78 (40.4), CG 90 (48.1)	Either traditional rehabilitation or traditional rehabilitation extended with an add-on program tailored to individual needs. The add-on program comprised a self-management booklet, motivational interviewing in structured individualized goal planning, and 4 supportive follow-up phone calls after discharge HCPs = rheumatologist/physician, physiotherapist, occupational therapist and nurse, other health professionals - 6 and 12 months	HRQoL measured by the Patient Generated Index (range 0–100, where 0 = low). patient-reported health status (SF 36-V2 mental and physical index), self-efficacy (Arthritis Self-Efficacy Scale (ASES), pain, fatigue, global disease activity, and motivation for change with NRS scales	89 subjects (44 CG, 45 IG) completed the 24-month follow-up. There was no significant difference in adherence at 24 months between groups (mean for CG 4.01 [95% confidence interval (95% CI) 3.03, 4.99], mean for IG subjects 3.63 [95% CI 2.70, 4.56]; *P* = 0.57)
Blixen et al. (2004), Australia [42]	To investigate the utility of a nurse-run telephone self-management program	RCT pilot feasibility study	32 OA pts/IG 16 pts/mean (SD) age: CG 69.9 (5.9), IG 71.7 (6.3)/female CG 31%, IG 44% Educational level: CG/IG Grade 7–9: 6%/6% Grade 10–11: 6%/0% High school: 19%/25% College: 1–4 years: 56%/44% College graduate: 13%/6% Professional/graduate school: 0%/19%	IG: six weekly mailings of OA health education modules, a relaxation audio-tape, and six weekly 45-min follow-up telephone self-management sessions + three sets of telephone interviews conducted by trained nurse interviewers blinded, who did not participate in the patient self-management program - 6 months follow-up	Arthritis self-efficacy scale: pain, function, other symptoms related to arthritis CES-D scale: depression level Satisfaction: Likert scale Open-ended OA self-management questions: self-efficacy	The change in NRS pain (mean difference 0.4 unit [95% CI − 0.4, 1.3]) and in WOMAC function (1.8 [95% CI − 1.9, 5.5]) did not differ between groups at 6 months, with both groups showing clinically relevant improvements. Some secondary outcomes related to physical activity and exercise behavior favored PT + coaching at 6 months but generally not at 12 or 18 months. There were no between-group differences in most other outcomes
Camerini et al. (2013), Switzerland [58]	To evaluate the effectiveness of an Internet-based patient education intervention, which was designed upon principles of personalization and participatory design	Cross-sectional study	209 fibromyalgia pts/mean age was 49 years (SD 10.0)/95% female 95% of participants completed at least 8 years of school, of which 82% also reported to have a high school or a university degree	Patients completed an online questionnaire to assess patients’ use of the website, health knowledge, self-management behavior, and health outcomes - N/A	Patients’ use of the website, health knowledge self-management behavior, and health outcomes (through online questionnaire, ten multiple-choice questions adapted from the website of the Mayo Clinic, and Fibromyalgia Impact Questionnaire (FIQ)	A significant treatment effect of the add-on intervention on HRQoL was found on discharge (*P* = 0.03). No significant between-group differences were found after 6 or 12 months. Both groups showed positive changes in HRQoL following rehabilitation, which gradually declined, although the values remained at higher levels after 6 and 12 months compared with baseline values
Collinge et al. (2020), USA [59]	To determine longitudinal associations between an online health diary program (“SMARTLog”) use and functional impact of FMS	Cohort study	76 FMS patients/mean age of 47 (SD 12) years/99% female 70% (*n* = 53) with college graduation	Participants used “SMARTLog” to report symptom ratings, behaviors and management strategies used [5 min three or more times per week over several weeks] - Every subsequent 30 days of program use as “follow-ups” during an 11-month open-use	Functional impact of FMS- FIQ	100% compliance rate IG and no drop outs at 3-month interviews; 1 subject in each group was lost to 6-month follow-up. There were no significant differences in self-management between the groups. At 3 months, there were improvements in the intervention group (relative to baseline) on some outcome measures. Self-reported QoL was extremely high, with an overall mean score of 5.8 (SD 0.8) (on a scale of 1–7, with 7 representing maximum satisfaction)
Cuperus et al. (2015), The Netherlands [43]	To compare the effectiveness of a non-pharmacological multidisciplinary F2F self-management treatment program with a telephone-based program on daily function in patients with GOA	Single-blind RC superiority trial	147 GOA patients/IG 76/mean (SD) age (CG) 61 [8], (IG) 59 [8]/female IG = 85% and CG = 85% N/A	6 weeks non-pharmacological multidisciplinary F2F treatment program [7 group sessions] OR a 6 weeks telephone-based treatment program (2 group sessions + 4 telephone contacts) - Multidisciplinary team with physical therapist, occupational therapist, specialized rheumatology nurse, and dietician - 6 months and 12 months	- Daily function measured with the Dutch consensus HAQ-DI - HRQoL measured with the SF-36 pain with SF-36 bodily pain subscale - Fatigue with Checklist Individual Strength (CIS); patients’ specific activity limitations with patient specific complaints questionnaire (PSK) -Self-efficacy with General Self-Efficacy Scale (GSES) - Physical activity levels with the Short Questionnaire to Assess Health (SQUASH) enhancing physical activity	Results show that the usage of certain tools of the website—designed and personalized involving the end users—impacts patients’ health knowledge, which in turn impacts self-management, that ultimately lower the impact of FMS leading to better health outcomes
Dahlberg et al. (2020), Sweden [44]	To investigate the long-term pain and function outcomes of people with hip or knee OA participating in a digital self-management program	Longitudinal cohort study	637 pts with hip (*n* = 198) or knee (*n* = 301) OA/24-week sub-sample mean (SD) age knee 65 (± 9) hip 64 (± 8)/female: knee 69% and hip 70%/48-week sub-sample mean (SD) age knee 64 (± 9) hip 63 (± 9)/female: knee 73% and hip 77% N/A	The digital OA self-management and education program (text or video lessons with quizzes)—consisted of a digital, structured, and individualized treatment program about instructions for daily neuromuscular exercises appropriately adjusted to each patient in regard to degrees of complexity and difficulty. The program has been dispensed-for a total of 70 lessons over a 48-week period + continuous access to and dialogue with a PT through an encrypted chat function, and/or telephone - 24 and 48 weeks	Joint pain-physical function pain-NRS, 0–10 worst to best - physical function (30-s chair stand test (30CST), number of repetitions) - Adherence as percentage of completed activities per the pre-defined period (the cut-off of 70% for the initial 3 weeks	Statistically significant inverse associations were found over time between FIQ scores (baseline M61.6 ± SD 14.7) and the cumulative number of SMARTLogs completed (*p* < .001); and the cumulative number of SMART Profiles received (*p* < .001)
de Thurah et al. (2018), Denmark [29]	To test the effect of PRO-based tele-health follow-up for tight control of disease activity in patients with RA, and the differences between tele-health follow-up performed by rheumatologists or rheumatology nurses	Non inferiority RCT	294 recruited RA patients - 254 patients (86%)/mean (SD) age in years CG = 60.7 (± 11.1) - PRO-TR group: 60.5 (± 13.5) - PRO TN group: 61.6 (± 13.5)/female sex (%) CG = 70% - PRO TR group: 65% - PRO TN group: 72% N/A	PRO-based tele-health follow-up scheduled for a telephone consultation (predefined checklist) every 3–4 months—prior to each consultation, the patients answered a questionnaire through the generic configurable tele-PRO-system, AmbuFlex A consultation (nurse or rheumatologist) was scheduled if Flare-RA > 2.5 and/or the patients CRP > 10 mg/L Patients in the CG were seen in the outpatient clinic every 3–4 months by physicians - 52 weeks	- Disease activity by DAS28 - RA flare with Flare-RA Instrument total score (all 11 items) or a subscale for joint or general symptoms - Physical function (HAQ), QoL (EQ-5D), and self-efficacy	No differences in effectiveness between both treatment programs on the primary outcome or on secondary outcome measures, except for a larger improvement in pain in the F2F treatment group (group difference (95% CI): 1.61 (0.01, 3.21)). Within groups, significant improvements were observed on several domains, especially in the face-to-face group
Durst et al. (2020), Germany [60]	To evaluate whether instruction and guidance via a digital app is not inferior to supervision by a PT with regard to movement quality, control competence for physical training, and exercise-specific self-efficacy	RCT 2 × 2 crossover trial	54 participants (HOA) started the first training session (32 women, 22 men; mean age 62.4, SD 8.2 years) 40.7% academic education	The intervention consisted of two identical training sessions with one exercise for mobility, two for strength, and one for balance One session guided by a PT and the other was guided by a fully automated tablet computer-based app. Both interventions took place at a university hospital The washout phase between the two interventions was set to range between 3 and 5 weeks	Outcomes were assessor-rated movement quality, and self-reported questionnaires on exercise-specific self-efficacy and control competence for physical training	A 24-week sub-sample: pain NRS decreased monthly by -0.43 units and 30CST repetitions increased monthly by 0.76 repetitions
Fedkov et al. (2022), Germany [30]	To evaluate the efficacy and safety of the DHA in patients with inflammatory arthritis	Explorative feasibility cross-sectional monocentric study	17 patients (12 RA, SpA: 1 axSpA, 4 PsA)/mean age 40.5 ± 12.2 years/female 59.8% N/A	The authors assessed demographic parameters, treatment regimen, disease activity, and other patient-reported outcomes at baseline and after 4 weeks of DHA use added to standard care treatment	HRQoL by SF-36, disease activity with CDAI/SDAI/ASDAS/BASDAI/RADAI, and PtGADA (scored from 0 to 100 mm), PPAIN (scored from 0 to 100 mm), BMI, HAQ, BFI, and PHQ-9 were used to evaluate obesity, physical impairment, fatigue, and depression, respectively	- A 48-week sub-sample, pain decreased monthly by -0.39 units, and 30CST repetitions increased by 0.72 repetitions
Gossec et al. (2018), France [61]	To assess in RA patients, the efficacy on patient-physician interactions, of giving access to Sanoia® platform	Multi-center RCT	320 RA patients/159 Sanoia® platform/mean (SD) age 57.0 (12.7) years/female 79.1% University-level: whole group: 51.9%, IG = 53.5%, CG = 50.3%	IG: access to the Sanoia® platform an e-health platform developed to allow self-assessment of health and disease status, patients received a 30-min information/training call to help them set up their Sanoia® accounts CG: usual care (continuation of normal Internet use without Sanoia® access) - 12 months	-Perceived Efficacy in PEPPI-5 questionnaire - Number of accesses to Sanoia® -satisfaction with the platform (NRS 0–10) - RAID questionnaire and RAPID3 - Disability HAQ-DI (range 0–3)	No clinically relevant effects on the improvement of pain or function by any covariate (age, sex, index joint)
Guaracha-Basáñez et al. (2022), Mexico [31]	To compare the impact of two healthcare interventions, hybrid care modality and F2F consultation, on the PRO of RA patients, during the COVID-19 pandemic	Non-inferiority RCT	130 patients (IG: = 65, CG *n* = 65), were primarily middle-aged females (90.1%), long-standing disease, working (62.8%), receiving DMARDs (96.7%), and corticosteroids (61.2%) The sample has received (median, IQR) 12 [9–16] years of education	Patients were randomized to 6 months of F2F consultation or hybrid care modality (intervention period-1) and then the converse modality (intervention period 2)	The primary outcome was disease activity/severity behavior (RAPID-3). Additional outcomes were disability (HAQ-DI, QoL (World Health Organization quality of life questionnaire-brief version), adherence and satisfaction with medical care, and treatment recommendation	Non-inferiority was established for the DAS28 in both tele-intervention groups when compared to conventional follow-up. In the ITT analysis, mean differences in the DAS28 score between PRO-TR versus CG were − 0.10 (90% confidence interval) and − 0.19 between PRO-TN versus control
Hernández-Zambrano et al. (2022), Colombia [32]	To describe changes in level of therapeutic adherence, QoL and capacity for self-care agency, during the follow-up period of a group of patients linked to a non-F2F multidisciplinary consultation model during the SARS-CoV-2 pandemic	Descriptive cohort study	71 RA patients/mean age between 33 and 86 years with a median of 63/female 85.9% Elementary 46.5% High school 26.8% Technician 19.7% University 5.6% Postgraduate 1.4%	Questionnaires carried out through telehealth calls (synchronous telemedicine) by the research group, which was composed of health professionals who are experts in each area and in the application of these scales - 12 weeks	- Therapeutic adherence was assessed using Morisky Green Test - Quality of life was assessed using EuroQOL-5-dimensions-3-level-version - Self-care capacity was assessed using ASA-R Scale	No differences between tele-health interventions and conventional follow-up were found in any of the secondary outcomes
Hoving et al. (2014), The Netherlands [62]	To evaluate the feasibility of an e-health intervention in rheumatology practice for employees with RA who experience problems with work functioning	Feasibility cohort study	23 out of the invited 90 RA pts/mean age (SD): 48 [10] years/female = 78% N/A	3-month internet e-health program consisting of a self-management program using a three-step problem-solving strategy: [1] analyze work problems and opportunities; [2] identify solutions; and [3] work out a strategy (action plan) Support and personal feedback by a rheumatology nurse - 12 months	Feasibility outcome included satisfaction with the program, measured by several questions in the follow-up questionnaire about the website, contact with the nurse and the program as a whole Other feasibility outcomes included usefulness, suitability, website use, and work functioning were measured at baseline and/or 3 months using questionnaires, semi-structured interviews, and website data	- The app was found to be inferior to the PT in all outcomes considered, except for movement quality of the mobility exercise
Kennedy et al. (2017), Canada [63]	To evaluate two modes of delivery of an inflammatory arthritis education program (“Prescription for Education” (RxEd)) in improving arthritis self-efficacy and other secondary outcomes	Non-randomized, pre-post study	123 arthritis pts/IG *n* = 36/mean age years m(SD): IG 58.6 (13.3), CG = 56.8 (13.1)/female IG = 87.4%, CG = 91.7% Primary/elementary school or less: IG = 2.3%, CG = 0 Secondary school: IG = 38.4%, CG = 39.1% Post-secondary school: IG = 52.3% CG = 60.9%	The RxEd Telemedicine Program was delivered into two different modes (“Remote” (R) sites versus “In-person” (I) site). Participants completed self-report questionnaires at baseline (T1), immediately after the RxEd session (T2), and at 6-month follow-up (T3) – 6 months survey	Self-report questionnaires served as the data collection tool. Arthritis Self-Efficacy Scale (SE), previous knowledge (Arthritis Community Research and Evaluation Unit (ACREU) rheumatoid arthritis knowledge questionnaire), coping efficacy, and Illness Intrusiveness and Effective Consumer Scale	In contrast to the two strengthening exercises in different positions, movement quality of the balance exercise was close to non-inferiority. Exercise-specific self-efficacy showed a strong effect in favor of the physiotherapist. In terms of control competence for physical training, the app was only slightly inferior to the physiotherapist
Khan et al. (2020), USA [64]	To demonstrate that a digital therapeutic intervention, utilizing a mobile app that allows self-tracking of dietary, environmental, and lifestyle triggers, paired with telehealth coaching, added to usual care, and improves QoL in SLE patients compared with usual care alone	RCT	50 SLE pts/IG = 27/median age was 43/female: 96% N/A	IG: 16-week therapeutic weekly telehealth coaching sessions 20–30-min plus usual care CG: usual care alone The digital therapeutic technology has 3 key components: a smartphone app for the patient to track lifestyle activities (e.g., diet, sleep habits, physical activity, bowel movements) and symptoms; software that analyzes and organizes data; and a web portal that presents all patient data to the health coach - 16 weeks	HRQoL tools: FACIT-F, BPI-SF, and LupusQoL	Statistically significant improvements (*p* < 0.05) for HRQoL (increase SPI-SF36 by 23.6%) and disease activity (decrease of C-DAI and S-DAI by 38.4% and 39.9%)
Lambrecht et al. (2021), Germany [65]	To assess the quality of the self-management app Rheuma Auszeit using the validated uMARS and to evaluate the association between uMARS scores and patients’ characteristics	Observational descriptive study	126 RMDs pts/mean (SD) age 52 (14.1) years/female 60.3% N/A	RMDs pts were asked to test Rheuma Auszeit, evaluate its quality using uMARS and complete a paper-based survey evaluating the individual preferences, attitudes and e-health literacy	Quality of the self-management app—using the validated uMARS (User Version of the Mobile App Rating Scale) app quality assessment tool	Clinically significant improvement was showed for SF-36 (+ 14.4%), disease activity (RADAI: 5 to 15.9%), and depression (Patient Health Questionnaire: 9 to 13.5%)
Lawford et al. (2020), Australia [66]	To explore therapeutic alliance between PT and KOA pts during telephone consultations	Longitudinal descriptive study	84 KOA pts/mean (SD) age 62.3 (9.3)/female 64% < 3 years of high school: *n* = 5 (6%) 3 years or more of high school: *n* = 19 (23%) Some tertiary training: *n* = 21 (24%) Graduated from university or polytechnic: *n* = 24 (29%) Any post-graduate study *n* = 15 (18%)	All patients (including those in the control arm) received a telephone call from a nurse where they received general information and advice about OA IG: patients additionally received between 5 and 10 telephone consultation from PT over a 26-week period to devise goals and an action plan that involved a home-based structured strengthening exercise program and/or a physical activity plan - 26 weeks	Therapeutic alliance was measured using the Working Alliance Inventory–Short Form	The usability level was high, i.e., the mean mHealth Application Usability Questionnaire Score of 5.96 (max.: 7.0)
Lee et al. (2019), South Korea [67]	To evaluate the utility and efficacy of a real-time pain monitoring system using a wearable device, referred to as the PAAS, in patients with FMS	RCT: pilot study-cross over	24 FMS patients/IG = 13/mean (SD) age IG = 42.8 (7.2), CG = 41.7 (11.2)/100% female N/A	Patients in the PAAS group were provided with a wearable device and smartphone application, while the control group was provided with usual treatment Pain visual analogic scale examined by rheumatologists at baseline and after 3 months, and correlations between conventional pain VAS or PAAS VAS and clinical parameters were investigated - 3 months	Pain VAS, disease activity with FIQ, PtGA, depression index with BDI and QoL with 5D-5L-EQ5D	Of 320 RA patients mean (SD) PEPPI scores at baseline and 12 months were 38.6 (8.2) and 39.2 (8.0) versus 39.7 (7.3) and 38.8 (8.0) in the IG and CG, respectively (*P* = 0.01)
Lu et al. (2020), Taiwan [68]	To determine the long-term effect of CM for Taiwanese RA patients	Quasi-experimental study	96 RA patients/experimental group = 50	The experiment group received a series of health education sessions and follow-up telephone consultations over a 6-month period, while the control group received only routine care during the same period A comparison of CM effects at three times (before CM/T0, 3 days after CM/T1, and 6 months after CM/T2) was made using generalized estimating equations (GEEs) -	Arthritis Self-Efficacy Scale (ASES) and Taiwanese Depression Questionnaire (TDQ)	Although mean satisfaction with the platform was very high (1.46 [1.52])
Magnol et al. (2021), France [69]	To describe e-Health use by RA patients, and to identify associations between patient demographics and disease characteristics and the use of e-Health tools and assess their expectations of e-Health	Multicenter cross-sectional study	575 RA patients/mean age of 62 (SD 13) years/female 77.7%	RA patients completed an anonymous self-questionnaire, including demographic data, evaluating their eHealth use (i.e., access, support, frequency of use, type of use, and reason for use). The rheumatologist completed an independent medical questionnaire on disease characteristics, activity of rheumatoid arthritis, and treatments - N/A	Data were collected during a single visit through an anonymous patient self-questionnaire with sociodemographic data, medical data, and assessment of the use of eHealth tools (i.e., access, support, frequency of use, type of use, and reason for use)	Patients with RA can monitor their disease online and create effective techniques to identify flare-ups. Ease of use, data security, and physician recommendations were factors that encouraged the usage of e-Health
Miró et al. (2022), Spain [70]	To conduct a preliminary evaluation of a mobile-app-delivered cognitive behavioral treatment (CBT)-based intervention in helping adults self-manage fibromyalgia symptoms	Observational study	53 FMS patients/mean age (M [SD]) 49.81, [9.99] years/female 94% N/A	Participants were asked to report every morning and evening for a 7-day period, as the baseline. This period was also used to identify any problems participants had with using the app), post-treatment, and 3-month follow-up. The app includes 9 modules that provide information about FMS and its treatment (with [1] PDF files that can be read, [2] videos that can be watched, and [3] audios that can be listened to). The treatment unfolds over the course of 47 treatment days. Treatment duration is ultimately up to the user, because different modules are unblocked depending on the user’s progress - 3 months	Pain severity (NRS 0–10), anxiety symptoms (Spanish version) of the Patient-Reported Outcomes Measurement Information System short form for Anxiety (PROMIS Short Form v1.0—Anxiety 8a), depression symptoms (Spanish version) of the PROMIS short form for Depression (PROMIS Short Form v1.0—Depression 8a), fatigue (Silhouettes Fatigue Scale (SFS)), and sleep quality (Pittsburgh Sleep Quality Index (PSQI) Spanish version), satisfaction (NRS 0–10)	Fibroline’s use is thought to be linked to improvements in clinical symptoms, and it was created as a resource for FMS patients
Murphy et al. (2021), USA [71]	To examine short and longer-term effects of two occupational therapy interventions on hand disability	RCT	Thirty-two SSc patients/(16 in intensive; 16 in app alone)/mean age 52 years (DS 13.4)/female 72% High school education (%, *n*); 25% (*n* = 8) total; 25% (*n* = 4) intensive group; 25% (*n* = 4) alone group	Two 18-week interventions: intensive group, receiving eight weekly in-person occupational therapy sessions with app-delivered home exercises, or app alone group Participants randomized into app alone scheduled 8-week and 18-week assessments In-person sessions consisted of 60 min of treatment-preparatory thermal modalities, tissue mobilization using the Lymphatouch device (Lymphatouch LLC), and UE passive and active range of motion exercises - 18 weeks	QuickDASH hand disability; PROMIS scale total active hand motion and home exercise adherence: daily app engagement: whether participants opened the app each day; adherence quality was based on time spent viewing exercise videos	RAID changes did not differ between groups
Oldroyd et al. (2022), UK [72]	To use daily data collected via a smartphone app for characterization of patient-reported and symptom-based (using an a priori definition) flares in an adult myopathy cohort	Observational study	Twenty idiopathic inflammatory myopathy patients/mean age/female 65%	UK adults with an IIM answered PROMs daily via a smartphone app during a 91-day study. Daily symptom PROMs addressed global activity, overall pain, myalgia, fatigue, and weakness (on a 0–100 visual analogue scale). Patient-reported flares were recorded via a weekly app question. Symptom-based flares were defined via an a priori definition related to increase in daily symptom data from the previous 4-day mean	PROMs (VAS) for global activity, overall pain, myalgia, fatigue, and weakness	Patients had low disease activity (median Routine Assessment of Patient Index Data-RAPID 3: 2.7) and HAQ-DI score that translated into the absence of disability, while QoL was compromised. Differences in RAPID-3 behavior were below the non- inferiority margin. Results considered the order in which patients received the intervention and baselines scores, and extended to the PRO left
Östlind et al. (2021), Sweden [73]	To describe physical activity (PA) patterns and adherence to WAT-use during an intervention among participants of working age with hip and/or knee OA	Observational study	75 hip or knee OA/mean age [45–66 years] N/A	Participants monitored their PA in the Fitbit-app. An activity goal of 7000 steps/day was set. Steps and minutes in light (L), moderate, and vigorous (MV) PA were collected from the Fitbit. Self-reported joint function (HOOS/KOOS) was completed	- Average daily number of steps per week - Average daily minutes in LPA and MVPA per week - Adherence to using the Fitbit - Average total weekly MVPA - Self-reported function	No significant changes were found in adherence to treatment in the total group, which demonstrates that there was no decrease in adherence, but neither was there an increase- patients did not stop taking the medication when they were well (*P* = .029)
Paolucci et al. (2022), Italy [74]	To investigate whether telerehabilitation that provides physical and psychological support services of the mind–body techniques can affect the clinical profile and pain relief of FMS patients	Observational study	28 FMS patients/mean (SD) age 6.61(8.56) years/female 100%	Rehabilitation treatment: 8 sessions, 1/week, 1 h/each, through Zoom platform, with the aim to anchoring to a positive emotion; listen and perceive your “own” body; conscious breathing; improve interceptive awareness; and relax The evaluations were performed at T0 (baseline), T1 (after 8 weeks of treatment), and T2 (after 1 month of follow-up) - 1 month	Assessment of the physical distress and fear of movement for the Numeric Rating Scale (NRS); the Fatigue Assessment Scale (FAS); the Fear Avoidance Belief Questionnaire (FABQ); with measures of physical and mental disability for the Fibromyalgia Impact Questionnaire (FIQ); the 12-Items Short Form Survey; the Resilience Scale for Adults and the Coping Strategies Questionnaire-Revised	In self-care capacity, there were significant improvements in five dimensions (*P* < .05), without significant differences in the global score—satisfaction = 4.6 out of 5
Pelle et al. (2021), The Netherlands [45]	To document the use and usability of the Dr. Bart app and its relationship with healthcare utilization and clinical outcomes in people with knee/hip OA	Retrospective observational study	214 knee and hip OA patients	Used backend data for the first 26 weeks of use by the intervention group (*N* = 214) of an RCT examining the effectiveness of the Dr. Bart app - 3 and 6 months	Self-management behavior, physical activity, health-related quality of life, illness perceptions, symptoms, pain, activities of daily living	- 95% of the pts were satisfied with the program
Pouls et al. (2022), The Netherlands [75]	To examine the effect on adherence to disease modifying anti-rheumatic drugs (DMARDs) in participants with RA of a serious game that targeted implicit attitudes toward medication	Multi-center RCT	110 RA patients	The intervention group played the serious game at will during 3 months, while having the usual care as the control group Game play data and online questionnaires were collected	Compliance Questionnaire on Rheumatology (CQR) Beliefs about Medicine Questionnaire (BMQ) Health Assessment Questionnaire (HAQ) Rheumatoid Arthritis Disease Activity Index (RADAI)	- 96% thought the program was useful
Rafiq et al. (2021), Malaysia [51]	To investigate the effectiveness of the lower limb rehabilitation protocol (LLRP) combined with mobile health (mHealth) applications on knee pain, mobility, functional activity and activities of daily living (ADL) among overweight and obese KOA patients	Single-blind RCT	114 knee OA patients	1. Rehabilitation group with mHealth (RGw-mHealth): LLRP + instructions of daily care (IDC) combined with mHealth intervention 2. Rehabilitation group without mHealth (RGwo-mHealth): LLRP + IDC intervention 3. Control group (CG): IDC intervention only All three groups were also provided leaflets explaining about their intervention	- Knee pain: Western Ontario and McMaster Universities Osteoarthritis Index score - Mobility: Timed up and go (TUG) test - Functional activity: patient-specific functional scale (PSFS) - ADL: Katz Index of independence in ADL scores	- 91% would recommend the program to other working RA patients
Rini et al. (2015), USA [46]	To reduce barriers that currently limit access to PCST (pain coping skills training)	Multi-center, single-blind RCT	113 hip or knee OA patients with pain	OA patients were randomized to a group completing PainCOACH [8 modules over 8 to 10 weeks] or control group with an assessment-only -	Osteoarthritis pain, pain-related interference with functioning, pain-related anxiety, self-efficacy for pain management, and positive and negative affect	All patients at least partially completed the assignments in step 1 and 12 patients completed at least one assignment in step 3
Rodríguez Sánchez-Laulhé et al. (2022), Spain [76]	To assess the short- and medium-term efficacy of a digital app (CareHand), together with educational and self-management recommendation, compared with usual care, for people with RA of the hands	Single-blind RCT	36 patients [58 hands] with RA/IG 26/58/mean age [43–78 years]/female 61%	IG: CareHand app, consisting of tailored exercise programs, and self-management and monitoring tools CG: written home exercise routine and recommendations, as per the usual protocol provided at primary care settings Both interventions lasted for 3 months [4 times a week] - Follow-up	- Hand function: Michigan Hand Outcome Questionnaire (MHQ) - Pain and stiffness intensity (visual analogic scale), grip strength (dynamometer), pinch strength (pinch gauge) - Upper limb function: shortened version of the Disabilities of the Arm, Shoulder, and Hand questionnaire	DAS averaged 2.8, and 12 participants showed remission (DAS < 2.6) at baseline
Rudin et al. (2020), USA [77]	To demonstrate that self-tracking technology, analytics, and telehealth coaching through a platform are useful to identify and remove possible dietary and/or other lifestyle triggers of SLE	Observational study	18 SLE patients	Intervention: 15-min on-boarding phone call with their health coach; then, participants were asked to start uploading pictures of all of their food intake to the app (for 12-week program period). Patients were instructed to continue compliance with usual care from their medical provider. Five to seven days later, the participant received a 45–60-min initial session with their health coach asking questions about their food intake, activity, environment and body functioning, and how these varied due to daily, weekly, monthly or yearly events - Follow-up of 12 weeks	HRQoL	Both groups (R and I) showed immediate effect (improved arthritis SE, mean change (95%CI): R 1.07 (0.67, 1.48) and I 1.48 (0.74, 2.23) after the program that diminished over 6 months (mean change (95% CI): R 0.45 (-0.1, 0.1) and I 0.73 (-0.25, 1.7)
Salaffi et al. (2015), Italy [78]	To assess the efficacy of a multicomponent intervention and evaluate the feasibility and user acceptance of an internet-based home telemedical surveillance system for the evaluation of pain and other key health outcomes in FMS patients	RCT	76 FMS patients/mean age multicomponent treatment 48.3 ± 11., control group 49.6 ± 12.3/female 100% IG: primary school 30.5%, middle school 50%, high school/university 19.5 CG: primary school 25%, middle school 58.3%, high school/university 16.7	Multicomponent treatment group consisted in an outpatient program of 24 twice-weekly sessions of combined aerobic, muscle strength training exercises and education - 3 months	FIQR FAS	For each of the secondary outcomes, both groups showed similar trends for improvement over time
Seppen et al. (2022), The Netherlands [34]	To assess the safety (non-inferiority in the DAS28-ESR) and efficacy (reduction in number of visits) of patient-initiated care assisted using a smartphone app, compared to usual care	RCT	103 RA patients/IG mean (SD) age 58 [13] years; CG mean (SD) age 57 [11] years/female 58%	IG: app-supported patient-initiated care with a scheduled follow-up consultation after a year CG: usual care - Follow-up 12 months	- DAS28-ESR score after 12 months - Mean number of consultations with rheumatologists FACIT-F BPI-SF-Pain Interference Lupus QoL-Pain Lupus QoL-Emotional Health Lupus QoL-Fatigue BPI-SF-Pain Severity Lupus QoL-Physical Health Lupus QoL-Burden to Others	In PP analysis, the group achieved significantly greater improvement than the control group in 9 of 11 domains: FACIT-F (*P* < .001), BPI-SF-Pain Interference ( *P* = .02), Lupus QoL-Planning (*p* = .004), Lupus QoL-Pain (*P* = .004), Lupus QoL-Emotional Health (*P* = .02), and Lupus QoL-Fatigue (*P* < .001) were significant when controlling for multiple comparisons; BPI-SF-Pain Severity (*P* = .049), Lupus QoL-Physical Health (*P* = .049), and Lupus QoL-Burden to Others (*P* = .04) were significant at an unadjusted 5% significance level
Serlachius et al. (2019), New Zealand [79]	To examine differences in self-reported user engagement between a recommended gout app (treatment group) and a dietary app (active control group) over 2 weeks as well as to examine any differences in self-care behaviors and illness perception	RCT	72 gout patients/mean (SD) age 49[15] years/female 14%	Patients were randomized to use either Gout Central (*n* = 36), a self-management app, or the Dietary Approaches to Stop Hypertension Diet Plan (*n* = 36), an app based on a diet developed for hypertension, for 2 weeks Outcome measures were assessed at baseline and after the 2-week trial	Self-reported user engagement-Mobile Application Rating Scale for user (uMARS, scale: 1 to 5) Self-care behaviors (scale: 1–5 for medication adherence and diet and 0–7 for exercise) Illness perceptions (scale: 0–10)	The median uMARS subjective quality was average (median 3.0, IQR 1.0)
Skougaard et al. (2020), Denmark [35]	To investigate intra- and interrater reliability and agreement of joint assessments and DAS-CPR in RA patients and test the effect of repeated joint assessment training	Prospective Dual-country Cohort Study	314 RA patients/ mean age 55 ± 13.5/ Female 81% N/A	All patients received training on joint assessment at baseline; only half of the patients received repeated training A subset of patients was included in an appraisal of interrater reliability and agreement comparing joint assessments completed by patients, healthcare professionals (HCP), and ultrasonography. Agreement was assessed using Bland–Altman plots - 2 months	Assessment of 28 tender and/or swollen joints was performed by patients 5 times on 4 different days over a period of 2 months For investigation of interrater reliability and agreement, 2 joint assessments were completed within a week at 2 occasions with an interval of 2 months, resulting in a total of 4 joint assessments Completion of the Health Assessment Questionnaire and VAS was done at all visits	e-Health literacy was significantly correlated with functionality uMARS subdomain ratings (Rho = 0.18; *P* < 0.042). Preferred time of app usage significantly correlated with engagement (*P* < 0.024), functionality (*P* < 0.029), total uMARS score (*P* < 0.017), and subjective quality score (*P* < 0.017)
Song et al. (2022), China [36]	To examine the effects of a theory-based educational delivered through WeChat on disease knowledge, self-efficacy, exercise adherence, and health outcomes in Chinese AS (Ankylosing Spondylitis) patients	Single-blinded RCT-parallel group	118 AS patients/mean age 29.9 (8.23) years/female 21.8% Junior high school or below 25.4%, Senior high school 22.0%, College or above 52.5%	Participants in the control group received standard care. The intervention group received the health belief model (HBM)-based educational intervention, consisting of 4 individual educational sessions and educational information sharing through WeChat, the predominant social networking app in China - N/A	Disease activity: Bath Ankylosing Spondylitis Disease Activity Index (BASDAI) Physical: Bath Ankylosing Spondylitis Functional Index (BASFI) Disease knowledge, self-efficacy, exercise adherence	At week 4, patients rated the overall alliance, and all 3 subscales, higher than therapists
Song et al. (2020), China [80]	To examine the effects of a tailored telehealth educational intervention on medication adherence and disease activity in discharged RA patients	RCT-parallel group un-blinded	92 RA patients/ 46 IG mean age 9.1 ± 7.58, CG: mean age 30.8 ± 8.82/female 21.2% Primary school or below 38 (49.4%)/IG 19 (46.3%) Junior high school 16 (20.8%)/IG 11 (26.8%) Senior high school 11 (14.3%)/IG 6 (14.6%) College and above 12 (15.6%)/IG 5 (12.2%)	IG: 4 educational sessions delivered through a telephone across a 12‐week intervention, including subject’s knowledge about disease; treatment goals; the importance of taking medication correctly; side effect management; remembering to take medication CG: received usual care including discharge instructions - 24-week follow-up	Medication adherence: Chinese version of Compliance Questionnaire Rheumatology (CQR). Disease activity: ESR, CRP and DAS28	For patients, the variables of living with others, consulting with a therapist with no previous experience delivering care remotely, having more telephone consultations, and having a higher self-efficacy were associated with greater alliance ratings
Song et al. (2021) China [81]	To evaluate the effects of the WeChat-based educational intervention on depression, HR- QoL, and other clinical outcomes in AS patients	RCT- single-blind, parallel-group	118 AS patients/59 pts/ mean age M ± SD 29.93 ± 8.23/female 21.2% ALL: Junior high school or below 25.4%, senior high school 22.0%, college or above 52.5% IG: Junior high school or below 25.4%, senior high school 20.3%, college or above 54.2% CG**:** Junior high school or below 25.4%, senior high school 23.7%, college or above 50.8%	Participants in the intervention group received the 12-week educational intervention delivered by WeChat and standard care (basic knowledge, exercise, medication, daily life management, psychological support, and self-assessment) The control group received standard care at hospital (basic health advice and brief guidance on medication and exercise, no access to educational program) - 12 weeks	Medical Outcomes Study Short Form 36-item Health Survey Beck Depression Inventory-II: BDI-II	The 12-week educational intervention through WeChat improved depressive symptoms and HRQoL in Chinese patients with AS. Thus, this intervention can be integrated into the current routine care of AS patients
Tore et al. (2022), Turkey [47]	To compare the effects of tele-rehabilitation Vs home-based exercise programs for KOA	RCT	48 KOA patients/24 IG/mean age IG:55.87 ± 7.24, CG: 55.79 ± 6.7/female 89.6% Primary school 52.1%, middle school 4.2%, high school 48%, university16.6%, postgraduate 2.1%	Telerehabilitation group: exercises via video conference simultaneously, accompanied by a physiotherapist CG: patients were given a brochure showing and explaining how to do each exercise - N/A	International Physical Activity Questionnaire Short Form (IPAQ-SF), (HADS), TAMPA Kinesiophobia Scale (TKS), Fatigue Severity Scale (FSS) twice before and after 8-week treatment, and (QUIPA) and Exercise Adherence Rating Scale (EARS) after treatment only	After the 8-week follow-up, telerehabilitation group demonstrated better 30 CST, IPAQ-SF, KOOS, QUIPA, treatment satisfaction, and total and C subscale of EARS scores increment and greater NRS, HADS, TKS, and FSS score reduction than the control group. Thus, telerehabilitation is superior to self-management
Tyrrell et al. (2016), UK [82]	To evaluate an e-Health tool, the AS Observer, specifically designed to monitor symptoms, QoL and physical activity in AS, in terms of patient experience and suitability in generating data for epidemiological studies	Observational pilot study	223 AS patients/mean (SD) age years 50.2 (13.9)/female 39.0% N/A	The AS Observer was designed to enable weekly monitoring of AS symptoms and exercise using a web-based platform. AS pts provided baseline data and completed online weekly data entry for 12 weeks (e.g., BASDAI, IPAQ). Panel data analysis with fixed effects models investigated associations between variables. Activity type data and exit questionnaires were subjected to qualitative thematic analysis - N/A	Disease activity: Bath Ankylosing Spondylitis Disease Activity Index (BASDAI) Physical activity: International Physical Activity Questionnaire (IPAQ)	82.2% of the participants had access to e-Health through a computer (86.1%), tablet (40.2%), or smartphone (47.3%). Of these, 36.4% (170/467) used the internet for health in general, and 28.7% (134/467) specifically for RA-related reasons. All these 134 patients used e-Health to learn about disease pathology, and 66.4% (89/134) of them used it as a tool to help monitor RA. Few patients (16/125, 12.8%) used tools for treatment reminders. Multivariate analysis showed that membership of a patient association, use of bDMARDs, and comorbidities are associated with e-Health use for RA
Umapathy et al. (2015), Australia [83]	To evaluate outcomes after usage of a Web-based resource (My Joint Pain) with tailored, evidence-based information and tools aimed to improve self-management of OA on self-management and change in knowledge	Quasi-experimental study	195 OA patients (users *n* = 104; nonusers *n* = 91)/mean (SD) age 60 [9] years/female 77.9%	The intervention provided participants with general and user-specific information, monthly self-assessments with validated instruments, and progress-tracking tools, at baseline and 12 months - N/A 12 months follow-up?	Health Evaluation Impact Questionnaire (heiQ) for patient education and self-management strategies Osteoarthritis Quality Indicator (OAQI) 17 items to assess appropriateness of care received	Data showed significant improvements in pain severity (*p* = 0.007), anxiety (*p* = 0.011) and depressive symptoms (*p* = 0.001) from pre-treatment to post treatment. Improvements were generally maintained, although there was some decrease in the outcomes from post-treatment to the 3-month follow-up. Most participants reported that they were very satisfied with the app (highly satisfied (39%) or satisfied (52%) with the app)
Vallejo et al. (2015), Spain [84]	To explore the effectiveness of Internet-delivered cognitive-behavioral therapy (iCBT) in treating FMS compared with an identical protocol using conventional group face-to-face CBT	RCT	60 FMS patients/IG/mean (SD) age 51.55 (9.87) years/female 100%	10 weeks of treatment: (a) The waiting list group (b) The CBT group (c) The iCBT group - Follow up 3–6-12 months	Impact of FMS on daily functioning: FIQ Psychological distress, depression, and cognitive variables, including self-efficacy, catastrophizing, and coping strategies	There were no significant between-group effects on QuickDASH (P = 1.0). Within groups, the Intensive group had the largest improvements after 8 weeks (*P* = 0.03), but then lost gains from 8 to 18 weeks, while the App Alone group had modest improvements from baseline to 8 weeks, but then continued to improve
Van Der Vaart et al. (2014), The Netherlands [85]	To measure the use, satisfaction and impact of a web portal which provides RA patients home access to their electronic medical records (EMR)	Pre-post study	360 RA patients/mean (SD) age 62(13.2) years/female 65%	To measure the use, impact of the portal, patients’ satisfaction with care, trust in their rheumatologist, self-efficacy in patient-provider communication, illness perceptions, and medication adherence satisfaction of a web portal with home access to the EMR -	- Health literacy level -Internet use (access, quantity of use and self-perceived skills) - Self-efficacy in patient-provider communication - Illness perception with the Revised Illness Perception Questionnaire - Medication adherence with Morisky Medication Adherence scale (MMA) - Self-efficacy in patient-provider communication with PEPPI - Patients’ satisfaction with care with QUOTE - Trust in their rheumatologist with Trust In Physicians short form	Of completers, 50% had clinically meaningful improvement on QuickDASH in the intensive group and 64% had improvement in App Alone. Overall median sample adherence was 49%, meaning that participants engaged with the App almost half of all study days
Wang et al. (2020), Australia [86]	To evaluate the effects of the updated version of the “My Joint Pain” website, on health education and quality of care over 12 months	Quasi-experimental study	277 patients 122 OA patients (users *n* = 35, non-users *n* = 87)/IG mean (SD) age 62.4 (8.3) years; CG mean (SD) age 60.3 (8.4) years/female 79%	Use of the updated version of the “My Joint Pain” website and its effect on health education and quality of care The changes from 12 to 24 months in the HEIQ were evaluated using a generalized linear model. The differences between users and non-users in the OAQI were evaluated using a chi-square test - Follow-up	Health Education Impact Questionnaire (HEIQ) OA Quality Indicator (OAQI) questionnaire	Patient-reported flares occurred on a median of 5 weeks (IQR 3, 7) per participant, out of a possible 13. Fatigue accounted for the most symptom-based flares and myalgia the fewest. Symptom-based flares typically resolved after 3 days, although fatigue-predominant flares lasted 2 days. The majority (69%) of patient-reported flare weeks coincided with at least one symptom-based flare
William et al. (2010), USA [87]	To assess incremental utility of adding an Internet-based exercise and behavioral self-management program for FMS	RCT	118 FMS patients/IG 59 patients/mean age M ± SD 50 ± 11.5 years/female 95% 18% high school, 40% college training, 30% college degree, 12% college graduation	Web-Enhanced Behavioural Self-Management (WEB-SM): website entitled “Living Well with Fibromyalgia” contained 13 modules-constructed for this study and translated content from traditional face-to-face cognitive-behavioral therapy for FMS into an educational self-help format	Pain: The Severity Scale of the Brief Pain Inventory Physical Functional: the SF-36 Physical Functioning Scale; Fatigue: MFI Sleep: MOS sleep scale Anxiety: STPI; depressive symptoms: CES-D Patient global impression of improvement: IMMPACT	Participants walked on average 10,593 (SD 3,431) steps/day, spent 248.5 (SD 42.2) minutes in LPA/day, 48.1 (SD 35.5) minutes in MVPA/day, 336.0 (SD 249.9) minutes in MVPA/week and used the Fitbit for an average of 88.4% (SD 11.6) of the 12-week period
Yuan SLK et al. (2020) Brazil [88]	To assess the effects of ProFibro app for 6 weeks compared to a traditional paper book of similar content to improve HRQoL, symptoms, and self-care in individuals with FMS	Single-blind, parallel trial RCT	40 FMS patients/IG: 20pts/mean age 43 ± 10.1 M ± SD/female 97.5% IG: secondary 25%, university 75% CG: primary 10%, secondary 40%, university 50%	6 weeks intervention (APP or book): aiming at patient education through animation; self-monitoring with the FIQR; sleep strategies with guided imagery relaxation technique, stimulus control therapy, and sleep hygiene; scheduling function to facilitate the planning of daily routine; graded exercise program with aerobic, stretching, and strengthening exercises; a diary for the practice of gratitude; an eBook with nine chapters on family adjustments; and hints through notifications - N/A	QoL: Revised Fibromyalgia Impact Questionnaire Severity of FMS: WPI and SS Pain: VAS Self-care: Self-Care Agency Scale	86.7% took > 7000 steps/day and 77.3% spent > 150 min in MVPA/week
Zhang et al. (2021), Switzerland [89]	To develop a mobile health (mHealth) tool for the remote self-assessment and monitoring of SSc-Dus	RCT	21 SSc patients with digital ulcers/ IG mean (SD) age 47[11] years; CG mean (SD) age 52[9] years/female 47%	SSc–DU pts to 2 device groups (basic and feedback) to self-document their DU at home over 8 weeks The color checker detection ratio (CCDR) and color checker sharpness (CCS) were compared between the 2 groups. The authors evaluated the feasibility of the mHealth tool by analyzing the usability feedback from questionnaires, user behavior and timings, and the overall quality of the wound images - Follow-up	- Color checker detection ratio (CCDR) - Color checker sharpness (CCS) - Patient satisfaction	The mHealth tool was useful in the daily routine of patients who were satisfied with the tool. The feedback group showed significantly higher (*p* < .001) CCS compared to the basic group
Zhao &. Chen (2019), China [90]	To explore the effectiveness of a health education program by telephone follow‐up on the self‐efficacy of RA patients	RCT	92 RA patients/mean (SD) age 55.5 (10.6) years/female 71.7%	IG: accepted health education by telephone follow‐up four times after the patients were discharged CG: only accepted telephone follow‐up once after they were discharged Self‐efficacy was measured by the use of RASE scale- data were collected at discharge, 12 and 24th week after discharged - Follow-up	Rheumatoid Arthritis Self‐Efficacy Questionnaire (RASE)	Tele-rehabilitation reduced physical and mental distress, fear, and disability (*p* < 0.001). Resilience and coping ability were less affected by the rehabilitative treatment

Legend: *AxSpA* axial Spondilo Arthritis, *BFI* brief fatigue inventory, *BMI* body mass index, *BPI-SF* Brief Pain Inventory-Short Form, *CG* control group, *CRP* C reactive protein, *DHA* Digital Health Application, *DMARDs* disease-modifying anti-rheumatic drugs, *EQ-5D* EuroQoL five dimension, *FACIT-F* Functional Assessment of Chronic Illness Therapy-Fatigue, *F2F* face to face, *FIQ* Fibromyalgia Impact Questionnaire, *FMS* fibromyalgia syndrome, *GOA* generalized osteoarthritis, *HAQ* Health Assessment Questionnaire, *HAQ-DI* Health Assessment Questionnaire Disability Index, *HCP* health care professional, *HRQoL* Health Related Quality of Life, *IG* intervention group, *KOA* knee osteoarthritis, *OA* osteoarthritis, *NRS* Numeric Rating Scale, *PAAS* Pain Assessment and Analysis System, *PEPPI* perceived efficacy in patient-physician interactions, *PHQ-9* Patient Health Questionnaire-9, *PPAIN* Patient’s Global Assessment of Pain Intensity, *PtGADA* Patient’s Global Assessment of Disease Activity, *PRO* patient reported outcome, *PT* physiotherapist, *QoL* Quality of Life, *RA* rheumatoid arthritis, *RAID* rheumatoid arthritis impact of disease, *RAPID 3* Routine Assessment of Patient Index Data 3, *RCT*, randomized controlled trial, *SF 36* Short Form 36 Health Survey, *SLE* systemic lupus erythematosus, *SpA* Spondilo arthritis, *SSc* systemic sclerosis, *SUA* serum uric acid, *PCST* pain coping skills training

**Table 4:** Data extraction for qualitative studies and mixed method studies

Author (year) country	Study aim	Study design	Patients/sample size/sex F%/age (M + SD)	Intervention	Outcomes measured and tools	Results
De Vries et al. (2017), The Netherlands [91]	To explore which patient-, intervention-, and environment-related factors are determinants of adherence to the online component of e-Exercise	Mixed method Semi-structured interview	109 participants with hip or knee OA/70% female, median age 60 years [51–79]	90 analyzed participants; 81.1% were evaluated for at least 8 weeks. Adherence was highest for participants with middle education, 1–5-year OA duration, and participants who were physiotherapist recruited	Adherence was defined as the number of online evaluated weeks. Next, semi-structured interviews on factors related to adherence to the online component were analyzed	The 10 analyzed interviews revealed that sufficient Internet skills, self-discipline, execution of the exercise plan, the intervention’s usability, flexibility, persuasive design, added value, and acceptable required time, and research participation were linked to favorable adherence
Fernon et al. (2016), Australia [92]	To identify useful eTool features and patient perspectives of using technology to manage their health	Semi-structured focus group	13 gout patients/mean age 60 years–median age 63 years/female 8% The highest level of education completed ranged from primary school (*n* = 2) to postgraduate study (*n* = 2)	Four semi-structured focus group sessions were held involved group discussions of potential e-Tool features and critiquing disease self-management websites and applications. Focus group sessions were audio-taped, transcribed, and analyzed by two independent researchers	Participants were open to using a supportive gout self-management e-Tool and identified a number of potentially helpful features, including educational material, serum uric acid monitoring, and medication reminder alerts	Participants were likely to use a supportive gout self-management eTool, identifying helpful features such as educational material, serum uric acid monitoring, and medication reminder alerts
Hinman et al. (2017), Australia [48]	To explore patients’ and physical therapists’ experience with Skype™ for exercise management of knee OA -	Grounded theory study	12 patients with OA, aged 62 years on average, half (*n* = 6) were female, and 8 physiotherapists (50% female)	12 purposively-sampled KOA patients who received for 3 months’ physical therapist-prescribed exercise over Skype™ and exercise equipment via courier (ankle weights, resistance exercise band, booklet with exercise instructions) and were assisted over the telephone by researchers to set up Skype™. Exercises were demonstrated by the therapist, and the participant performed the exercise, while the physical therapist watched. At subsequent sessions, exercises were reviewed and progressed Patients and therapists who delivered the intervention were interviewed about their experiences. The 2 semi-structured interviews for each participant were audio-recorded and transcribed. Two investigators undertook coding and analysis using a thematic approach	Patients’ and physical therapists’ experience with Skype™ for exercise management of knee OA	Six themes arose from both patients and therapists Structure: (i) technology (easy to use, variable quality, set-up assistance helpful) and (ii) patient convenience (time efficient, flexible, increased access) Process: (iii) empowerment to self-manage (facilitated by home environment and therapists focusing on effective treatment) and (iv) positive therapeutic relationships (personal undivided attention from therapists, supportive friendly interactions) Outcomes: (v) satisfaction with care (satisfying, enjoyable, patients would recommend, therapists felt Skype™ more useful as adjunct to usual practice) and (vi) patient benefits (reduced pain, improved function, improved confidence and self-efficacy). A seventh theme arose from therapists regarding process: (vii) adjusting routine treatment (need to modify habits, discomfort without “hands-on,” supported by research environment)
Muehlensiepen et al. (2022) Germany [93]	To investigate patient and physician experiences, perceived drawbacks, and benefits of using an ePRO web-app (ABATON RA) to digitally support SDM and T2T	Qualitative study	10 RA patients/mean age was 51 (range 27–73)/female 50%)	A qualitative study embedded in a multicenter randomized controlled trial (RCT) interviews with RA patients and physicians that were subsequently analyzed using deductive-inductive qualitative content analysis	Patient and physician experiences, perceived drawbacks and benefits of using an ePRO web app	Between August 2021 and May 2022, interviews with ten RA patients and five physicians were completed. Three key themes emerged in the analysis: (i) App user experiences; (ii) perceived drawbacks of app-supported rheumatology care; and (iii) perceived benefits of app-supported rheumatology care. Continuous ePRO collection and a high level of standardization strained some RA patients. Certain ePRO seemed outdated and were hard to understand. Patients and physicians appreciated having an improved overview of disease activity, capturing disease flares and continuous remote monitoring. Paper- and time-saving were associated with using ePRO. Physicians feared to become too focused on ePRO data, stressed the lack of ePRO monitoring reimbursement and app interoperability. For RA patients and physicians, benefits seemed to outweigh observed drawbacks of the digitally supported SDM using ePRO. The software was easy to use and could lead to a better understanding of the individual disease course, resource allocation and treatment of rheumatoid arthritis
Najm et al. (2020) (European countries, the USA, Canada, and Australia) [94]	To explore the needs, experiences, and views of people diagnosed with RMDs on mHealth apps	Focus group-mixed method	424 RMDs patients/range age 45 to 54 years (122/424, 28.7%), female 86.8% Focus group: 6 RMDs patients	3/6 patients had used a self-management app at least once 424 RMDs patients were asked about their experience	Self-management, disease activity, communication with HP	Among 424 patients, half of the respondents were aware of the existence of apps to support self-management of their RMDs (188/355, 53%), with 42% (79/188) of them currently using such devices. Patients were mostly interested in an app to self-monitor their health parameters (259/346, 74.9%) and disease activity (221/346, 63.9%) or communicate directly with their health care provider (200/346, 57.8%) The use of existing apps was reported as time-consuming due to a lack of functionality. The need for bespoke apps was voiced by all participants
Pani et al. (2017), Italy [95]	To evaluate patients’ perspectives on the use of a system for home tele-rehabilitation, designed for subjects with low computer literacy suffering hand impairment due to RMDs	Mixed method study with semi-structured interviews	20 RMDs patients	After a clinical trial assessing device effectiveness, the PIADS, the QUEST, and the IPPA questionnaires were administered to evaluate the system’s impact on each patient’s life, and the results were correlated with clinical indices Patients were asked to continue self-administered rehabilitation with common objects. One year later, a semi-structured telephone interview gathered data on their experience	Psychosocial Impact of Assistive Devices Scale (PIADS), Quebec User Evaluation of Satisfaction with Assistive Technology (QUEST) and Individually Prioritised Problem Assessment (IPPA)	The system received a positive QUEST score (4.5 ± 0.3) and a modest PIADS score (0.84 ± 0.8) due to the small impact on adaptability and self-esteem. The IPPA (3.7 ± 3.4) revealed improvement in the ability to perform tasks considered important, which was significantly correlated (*r* = 0.60; *p* < 0.02) with the clinical Health Assessment Questionnaire (HAQ) index improvement. The interviews revealed a positive engagement effect, enhanced by the need to develop skills to be able to use the device (technological challenge) and by the perception of more attention by the medical staff. This may explain the significant dropout rate (80%) from the post-trial rehabilitation of the patients who used the device
Shewchuk et al. (2021), Canada [96]	To evaluate the overall usability, quality, and effectiveness of the mHealth app prototype for aiding knee OA self-management from the perspectives of patients with OA and HCPs	Mixed method with semi structured interviews and surveys	18 OA patients	Qualitative: patient and HCP perspectives were elicited on the likeability and usefulness of app features and functionalities and the perceived impact of the app on patient-HCP communication The quantitative assessment involved evaluating the app using usability, quality, and effectiveness metrics. Patient baseline assessments included a semi-structured interview and survey to gather demographics and assess the QoL and patient activation. Following the 6-week usability trial period, a follow-up survey assessed patients’ perceptions of app usability and quality and longitudinal changes in QoL and patient activation	Patient baseline assessments included a semi-structured interview and survey to gather demographics and assess the quality of life (European Quality-of-Life 5-Dimension 5-Level Questionnaire [EQ-5D-5L]) and patient activation (patient activation measure [PAM])	Interviews with patients and HCPs revealed overall positive impressions of the app prototype features and functionalities related to likeability and usefulness. Between the baseline and follow-up patient assessments, the mean EQ-5D-5L scores improved from 0.77 to 0.67 (*P* = .04), and PAM scores increased from 80.4 to 87.9 (*P* = .01). Following the 6-week evaluation, patients reported a mean System Usability Scale (SUS) score of 57.8, indicating marginal acceptability according to SUS cut-offs The mean number of goals set during the usability period was 2.47 (SD 3.08), and the mean number of activities completed for KOA self-management during the study period was 22.2 (SD 17.8) HCPs reported a mean SUS score of 39.1, indicating unacceptable usability
Vanderboom et al. (2014), USA [97]	To determine the feasibility of technology-enhanced monitoring that engages FMS patients using a mobile device	Mixed method with focus group and surveys	20 FMS patients	Patients used a mobile monitoring device for 1 week; nurses responded to patient e-mailed symptom reports on a daily basis For qualitative data, a 1-h focus group was audio-recorded, transcribed verbatim, and then analyzed using content analysis. All participants used a mobile phone in their daily lives; half used a smart phone	Participants’ current use of technology was measured through a seven-item survey developed by the investigator. The use of the monitoring device was measured through the Perceived Ease of Use (PEU), the Perceived Usefulness (PU) Questionnaires, and through the actual use of the monitoring device. The feasibility of study procedures was evaluated through focus group	Participants were interested in using a smart phone to monitor their health and to communicate with HCP Participants used the study mobile device an average of 5.2 days out of the 7 day study period. Most participants (80%) reported that monitoring symptoms using the device was easy to do 65% felt that using the device helped them to promptly address their symptoms

### Phase V: collating, summarizing, and reporting the results

Findings concerning telemedicine interventions for patients with rheumatic diseases were then summarized and structured to offer a comprehensive overview of the existing evidence in this domain. Additionally, an appraisal of the quality of the evidence for the included studies was conducted by applying the Critical Appraisal Skills Programme (CASP) tools for both quantitative and qualitative studies [27]. The total number of “yes” responses determined the quality of the studies, based on the established weight of the items. A CASP score > 7 indicates a study of good quality; a CASP score < 3 indicates a study of low quality. Each reviewer conducted an independent quality assessment. The CASP classifies the quality of evidence as three levels: high, moderate, and low. As stated for the screening process, disagreement on the assessment was solved by a third reviewer (SB). As for the mixed method study design, the mixed methods appraisal tool (MMAT) scale was used for quality assessment, as the CASP for mixed method studies is not available [28]. We do not present an overall score for mixed method studies, as the authors of the MMAT scale discourage to do so, instead, they advise to provide a more detailed presentation of the ratings of each criterion to better inform the quality of the included studies.

## Results

**Fig. 1:**
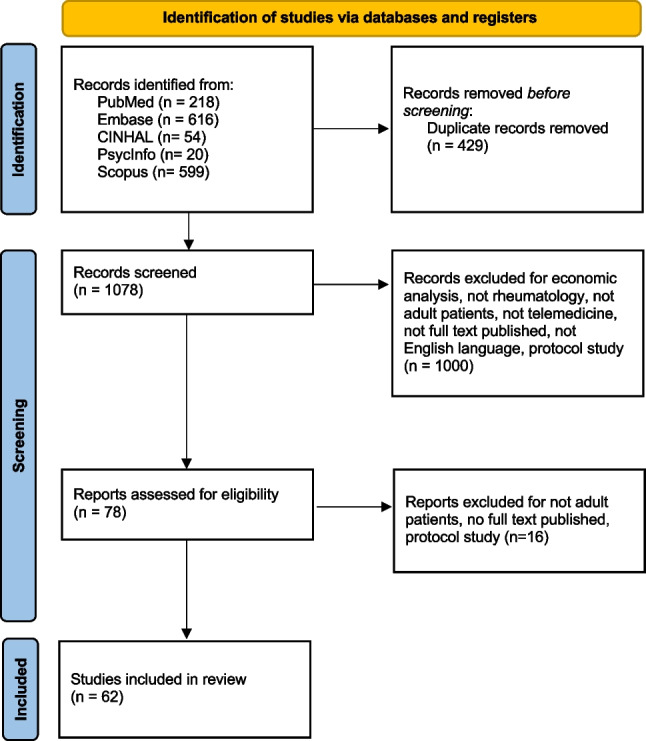
Flowchart of the records retrieved

Using our search strategy, a total of 1507 records were identified, and then using Endnote software, 1078 were identified as duplicates and removed (Fig. 1). Of these, 1000 were excluded based on title or abstract. For the 78 studies remaining, 16 were excluded following a full-text review for failing to meet other inclusion criteria. Thus, a total of 62 studies were ultimately included in this scoping review: 54 quantitative study designs, 4 qualitative study designs, and 4 mixed method study design. Tables 3 and 4 summarize the data extracted from the 62 included studies.

### Prevalence of RMDs in the selected studies

In the selected studies, the most represented RMDs are RA (29.0–18/62) and OA (27.4%–17/62); then, we have fibromyalgia syndrome (FMS) (16.1–10/62). As for other RMDs, four studies (6.5%) included patients with connective tissue diseases (CTDs) (two systemic lupus erythematosus (SLE) and two systemic sclerosis (SSc)), three studies included patients with gout (4.8%), three studies included patients with ankylosing spondylitis (AS) (4.8%), two studies included patients with inflammatory arthritis other than RA (3.2%), and 1 study included patients with idiopathic inflammatory myopathy (1.6%). The remaining four studies (6.5%) did not specify the rheumatic diagnosis of RMDs patients included.

### Country of the selected studies

**Fig. 2:**
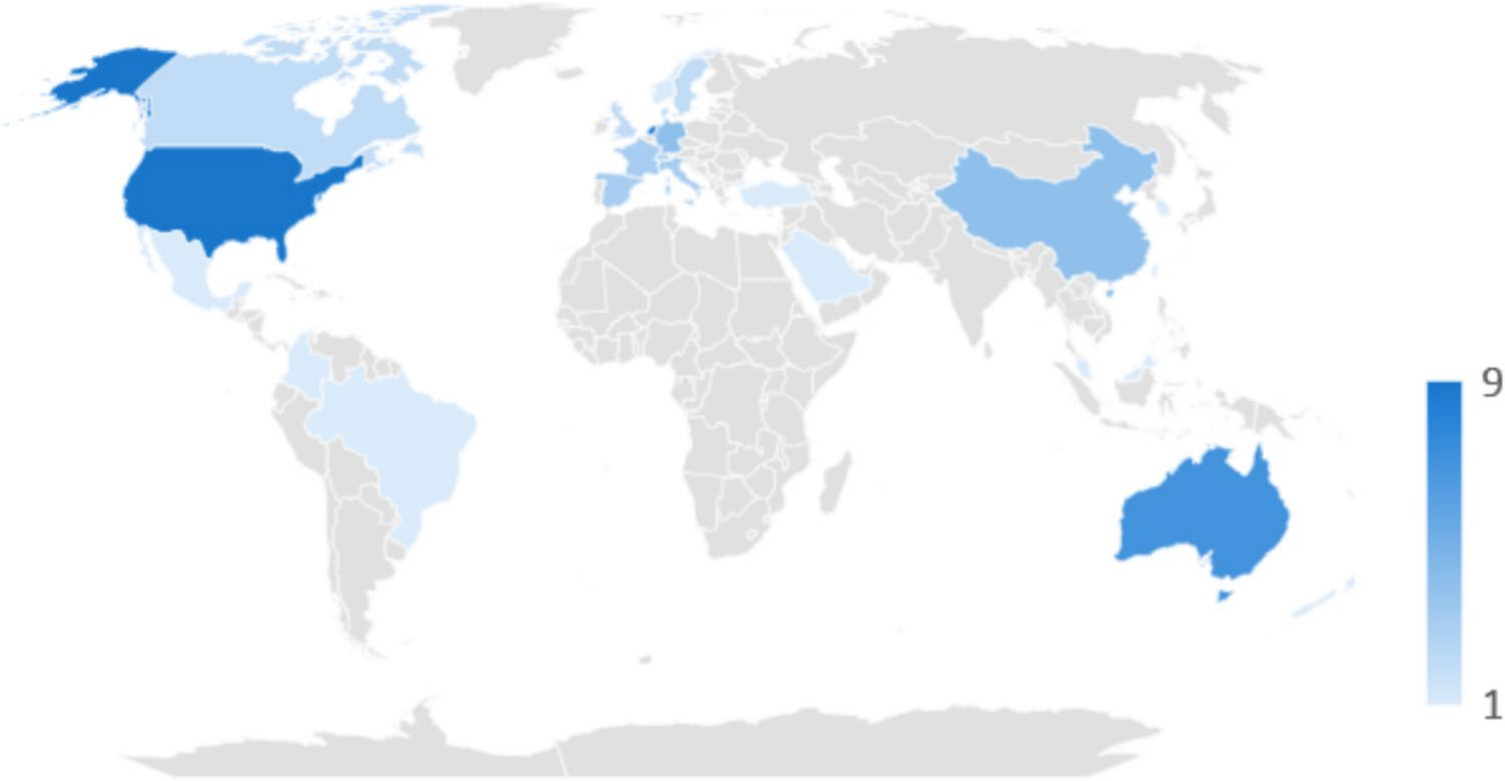
Geographical distribution of the studies included in the present scoping review

As for the country of publication of the included studies, 50% (31/62) was conducted in Europe; 14.5% (9/62) in United States of America and the same percentage in Asia; 12.9% (8/62) was conducted in New Zealand and Australia, while the remaining studies were conducted in South America or Saudi Arabia (Fig. 2).

### e-Health tools used in the selected studies

**Fig. 3:**
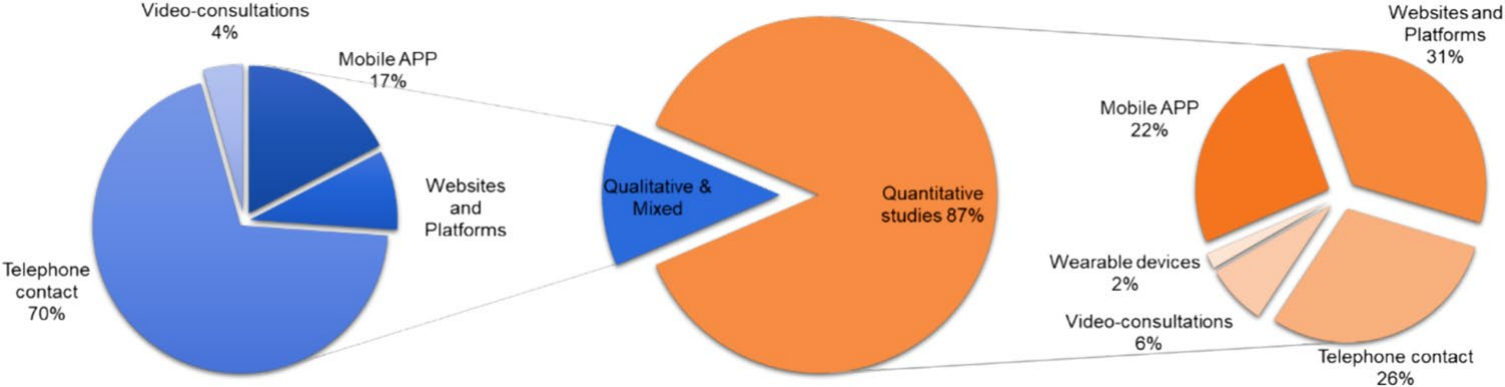
Prevalence of e-health tools used in the included studies, shown per type of study design

Multiple e-health tools were used in the studies included in the present review, namely 33.9% (21/62) used websites and online platforms, 29.0% (18/62) used mobile applications, 25.8% (16/62) used telephone contacts, 8.1% (5/62) used video-consultations, and only one study (1.6%) used wearable devices. The remaining one study included telemedicine in general, without specifying the tools or devices. In Fig. 3, devices used in the studies are represented following the study design, either qualitative or quantitative.

### Clinical outcomes reported in the selected studies

**Fig. 4:**
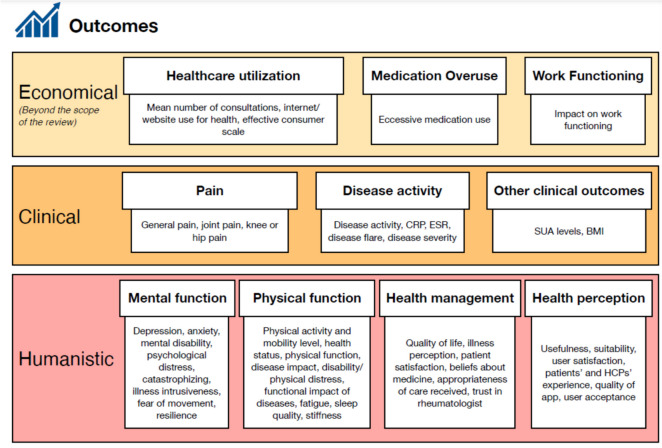
Outcome measures reported following the ECHOs classification

Pain, as a clinical outcome (i.e., general pain, joint pain, or knee pain), has been assessed in most of the studies, meaning that all the telemedicine tools except for those using video-consultations. Also, one study on mobile applications included pain assessment in the qualitative study design. Disease activity, as a clinical outcome (i.e., disease activity measured by the clinicians including erythrocyte sedimentation rate (ESR) and/or C-reactive protein (CPR) values, and disease flare and disease severity) was assessed with all kinds of telemedicine tools. Other clinical outcomes assessed were serum uric acid (SUA) levels and body mass index (BMI), measured with telephone contact and mobile applications, respectively (Table 5 and Fig. 4).

### Humanistic outcomes reported in the selected studies

As for the humanistic outcomes according to the ECHO framework, the authors agreed on including a comprehensive categorization of the outcomes classifying them as regarding the mental and physical function or the health management and health perception (Table 5 and Fig. 4).

**Table 5:**
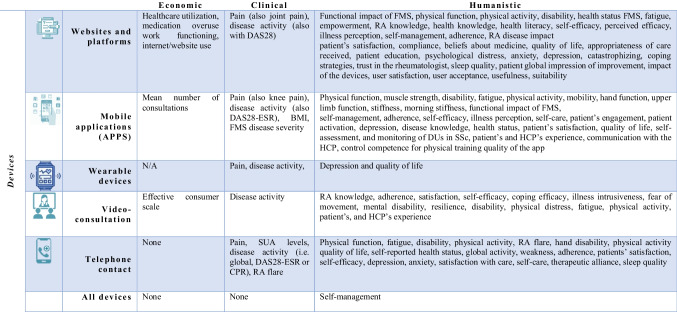
Economic, clinical, and humanistic outcomes of the included studies of the present review

Mental function included outcome measures as depression and anxiety, mental disability and psychological distress, catastrophizing, illness intrusiveness, fear of movement, and resilience. Physical function included outcome measures as the level of physical activity and mobility (i.e., general, hand, or upper limb function), health status and physical function, disease impact (i.e., RA and FMS), disability or physical distress, functional impact of the diseases (i.e., FMS), physical activity, and other outcomes such as fatigue, sleep quality, and stiffness. Health management included outcome measures such as disease knowledge (i.e., RA or FMS), treatment adherence, patient empowerment, activation and engagement, self-efficacy, self-care, self-management, coping strategies, health literacy, and patient education. One study included self-assessment and monitoring of Digital Ulcers (Dus) in SSc.

Health perception included outcome measures such as quality of life, illness perception, patient’s satisfaction, beliefs about medical treatment, appropriateness of care received, and trust in the rheumatologist. Lastly, other outcome measures related to the experience of the patients with the e-health tool used are included in some studies: In particular, they assessed usefulness, suitability, user satisfaction, patients’ and HCPs’ experience, quality of the app, and user acceptance.

### Quality assessment of the studies

We conducted the quality assessment of the included studies, as specified in the method section (Tables S6 to S12, Supplementary information). The three qualitative studies assessed were all of good quality. As for the quantitative studies, we assessed 15 observational/cohort studies with 5 good quality studies, 6 medium quality studies, and 4 low quality studies and 39 experimental/clinical trial with 14 good quality studies, 22 medium quality studies, and 3 low quality studies.

## Discussion

The present scoping review aimed at identifying the state of the art regarding the application of digital technology, in terms of devices used and in terms of PROs assessed in the rheumatology field. The outcomes of this scoping review helped us to address our original query and comprehend the current state of the art when it comes to using all telemedicine tools in the context of rheumatology, and particularly those that are linked to specific outcomes. To date, it is commonly recognized that information technology is important and should be integrated to traditional care and assistance models.

Regarding the studies included in this scoping review, the methodological variety of study designs is undoubtedly something worth reflecting on. The presence of quantitative, qualitative, and mixed method studies highlights the heterogeneity of the aspects that can be investigated in the field of telemedicine in rheumatology.

Moreover, most of the studies included in this scoping review were conducted in European countries, USA, and also Australia make our results generalizable to RMDs populations, clearly after tailoring them to the clinical context.

As for the disease prevalence, OA and RA are the most studied diseases among RMDs, followed by CTDs and FMS. Indeed, several studies addressed telemedicine for patients with RA and OA suggesting that healthcare facilities are more likely to invest in telemedicine for these conditions due to their high prevalence.

Regarding the outcomes assessed for RA, quality of life and disease activity are present in the majority of the studies [29–36]. In line with the literature, these are important outcomes to assess when dealing with arthritis as the disease affects the quality of life of RA patients by 93% and the ability to perform even the simplest daily gestures, such as opening a bottle, performing activities of daily life, climbing stairs, dressing, or washing by 85% [37].

In addition, disease activity is a pivotal parameter to include and monitor over time for all arthritis patients; in fact, specific questionnaires, that integrate inflammatory indices such as CRP or ESR in addition to patient-reported issues and health status, are widely used among the scientific and clinical community [38].

As for OA, most studies included further outcomes such as treatment adherence, in particular physical exercise and self-management, as these represent the core strategies when dealing with these patients [29, 39–48].

In the present review, the heterogeneity emerged from the included studies in terms of disease diagnoses, outcomes evaluated, and digital technologies used. Indeed, multiple tools of telemedicine, namely more than 1/3 of them used websites and online platforms, almost 1/3 used mobile applications, and the last 1/3 used telephone contacts. Video-consultations and wearable devices are less likely to be used when assessing or treating patients with RMDs.

Furthermore, most studies that compare e-health interventions with standard of care, or standard visits and assistance, found that the use of telemedicine produces either positive or non-inferior outcomes [42, 46, 47, 49–51]. These studies highlight the significance of both the patient’s adherence to the treatment plan and the monitoring provided by HCPs, regardless of the digital tool utilized, which represent fundamental outcomes for chronic inflammatory RMDs. Moreover, these factors are crucial for nursing practice, indeed, according to Gordon’s functional health patterns of health perception/management and activity/exercise, nurses, and HCPs in general, can plan interventions to promote treatment adherence while maintaining an appropriate quantity of physical activity [52].

When it comes to chronicity, self-management, and patient’s autonomy in managing their illness are generally encouraged by specific mobile applications, and nearly all findings indicate that the use of these e-tools has a positive effect on PROMs. Indeed, also other studies are addressing the need to define the role of digital tools regarding self-management in RMDs patients [53].

Essentially, these tools allow the patients to self-manage and monitor their condition, with direct access to relevant information and data about their disease, while encouraging patient’s autonomy.

When telephone contacts are used, findings from the review showed that patient outcomes are favorable specifically regarding disease activity and disease management. Indeed, compared to websites and smartphone apps, telephone contact is more likely to offer inclusivity for patients with lower digital health literacy. This aspect and related factors have not received enough research attention, yet technology resources are today widely available for patients, thus evaluating that the patients’ digital health literacy level is mandatory and determine its casual factors [54].

To summarize, the comprehensive disease monitoring, consistent with the person-centered approach, is in accordance with the results of the review, especially when we look at the humanistic outcomes. In fact, dealing with mental and physical function, health management, and perceptions allow us to assure a comprehensive assessment required for RMDs.

In addition, it is critical to recognize and detect timely significant symptoms or flare-ups to refer patients to face-to-face consultations and prevent further complications.

Indeed, before implementing digital health interventions, a thorough evaluation of the patient in terms of clinical condition, health literacy level, and social context of life is required to provide international working groups’ protocols, recommendations, and guidelines.

### Strengths and limitations

This review has some strengths. First, diverse study designs provide a holistic understanding of telemedicine interventions. Second, valuable insights into improved disease management and patient-reported outcomes are highlighted. However, the clinical heterogeneity of tele-healthcare interventions and methodological bias can also constrain the evidence for effectiveness, but they support the personalization of clinical care paths.

### Implications for clinical practice and research

The review underscores the need to critically evaluate research quality and tailor care pathways in telemedicine for rheumatological conditions. While digital tools hold promise, challenges such as technological diversity and patient health literacy require attention.

Future studies should focus on integrating patient perspectives for effective implementation and person-centered care.

In conclusion, the present scoping review showed the heterogeneity of digital tools in the field of rheumatology, which underlines the difficulty in transferring to clinical practice the results of valid studies. Clearly, OA and RA are the most studied diseases among RMDs, and effective tele-rehabilitation models have been presented, along with the application of the tight control strategy that recently emerged to be necessary in disease monitoring to achieve disease remission. Future studies should focus on summarizing and producing clinical recommendations for RMDs and developing studies on other RMDs, such as CTDs or FMS.

### Supplementary Information

Below is the link to the electronic supplementary material.

Supplementary file1 (DOCX 141 KB)

## Data Availability

The datasets for this study can be requested to the corresponding author.
