# Supplementary material for: Evidence for telemedicine heterogeneity in rheumatic and musculoskeletal diseases care: a scoping review
Source: Clin Rheumatol. 2024 Jul 10;43(9):2721–63. doi: 10.1007/s10067-024-07052-w (PMC11330403; doi:10.1007/s10067-024-07052-w)
Supplement: Supplementary file 1 — Supplementary file1 (DOCX 141 KB) [file 10067_2024_7052_MOESM1_ESM.docx]

Supplementary Material

# Supplementary Tables

# *Table 6. Critical appraisal skills programme (CASP) results of included clinical trials*

***Table 7.*** *Critical appraisal skills programme (CASP) results of included observational studies*

***Table 8.*** *Critical appraisal skills programme (CASP) results of included qualitative studies*

***Table 9****. The following tables show the quality assessment for mixed method study with the Mixed Methods Appraisal Tool (MMAT), version 2018*

| **De Vries et al (2017)** | **Methodological quality criteria** | **Responses** | | | |
| --- | --- | --- | --- | --- | --- |
|  |  | Yes | No | Can’t tell | Comments |
| Screening questions (for all types) | S1. Are there clear research questions? | X |  |  |  |
|  | S2. Do the collected data allow to address the research questions? | X |  |  |  |
|  | *Further appraisal may not be feasible or appropriate when the answer is ‘No’ or ‘Can’t tell’ to one or both screening questions.* | | | | |
| 1. Qualitative | 1.1. Is the qualitative approach appropriate to answer the research question? | X |  |  |  |
|  | 1.2. Are the qualitative data collection methods adequate to address the research question? | X |  |  |  |
|  | 1.3. Are the findings adequately derived from the data? | X |  |  |  |
|  | 1.4. Is the interpretation of results sufficiently substantiated by data? | X |  |  |  |
|  | 1.5. Is there coherence between qualitative data sources, collection, analysis and interpretation? | X |  |  |  |
| 2. Quantitative randomized controlled trials | 2.1. Is randomization appropriately performed? | X |  |  |  |
|  | 2.2. Are the groups comparable at baseline? |  |  | X |  |
|  | 2.3. Are there complete outcome data? |  |  | X |  |
|  | 2.4. Are outcome assessors blinded to the intervention provided? |  | X |  |  |
|  | 2.5 Did the participants adhere to the assigned intervention? |  | X |  |  |
| 3. Quantitative non- randomized | 3.1. Are the participants’ representative of the target population? |  |  |  | N/A |
|  | 3.2. Are measurements appropriate regarding both the outcome and intervention (or exposure)? |  |  |  | N/A |
|  | 3.3. Are there complete outcome data? |  |  |  | N/A |
|  | 3.4. Are the confounders accounted for in the design and analysis? |  |  |  | N/A |
|  | 3.5. During the study period, is the intervention administered (or exposure occurred) as intended? |  |  |  | N/A |
| 4. Quantitative descriptive | 4.1. Is the sampling strategy relevant to address the research question? |  |  |  | N/A |
|  | 4.2. Is the sample representative of the target population? |  |  |  | N/A |
|  | 4.3. Are the measurements appropriate? |  |  |  | N/A |
|  | 4.4. Is the risk of nonresponse bias low? |  |  |  | N/A |
|  | 4.5. Is the statistical analysis appropriate to answer the research question? |  |  |  | N/A |
| 5. Mixed methods | 5.1. Is there an adequate rationale for using a mixed methods design to address the research question? | X |  |  |  |
|  | 5.2. Are the different components of the study effectively integrated to answer the research question? |  |  | X |  |
|  | 5.3. Are the outputs of the integration of qualitative and quantitative components adequately interpreted? | X |  |  |  |
|  | 5.4. Are divergences and inconsistencies between quantitative and qualitative results adequately addressed? |  | X |  |  |
|  | 5.5. Do the different components of the study adhere to the quality criteria of each tradition of the methods involved? | X |  |  |  |

***Table 10.*** *The following tables show the quality assessment for mixed method study with the Mixed Methods Appraisal Tool (MMAT), version 2018*

| **Vanderboom et al. (2020)** | **Methodological quality criteria** | **Responses** | | | |
| --- | --- | --- | --- | --- | --- |
|  |  | Yes | No | Can’t tell | Comments |
| Screening questions (for all types) | S1. Are there clear research questions? | X |  |  |  |
|  | S2. Do the collected data allow to address the research questions? |  |  | X |  |
|  | Further appraisal may not be feasible or appropriate when the answer is ‘No’ or ‘Can’t tell’ to one or both screening questions. | | | | |
| 1. Qualitative | 1.1. Is the qualitative approach appropriate to answer the research question? | X |  |  |  |
|  | 1.2. Are the qualitative data collection methods adequate to address the research question? | X |  |  |  |
|  | 1.3. Are the findings adequately derived from the data? | X |  |  |  |
|  | 1.4. Is the interpretation of results sufficiently substantiated by data? | X |  |  |  |
|  | 1.5. Is there coherence between qualitative data sources, collection, analysis and interpretation? | X |  |  |  |
| 2. Quantitative randomized controlled trials | 2.1. Is randomization appropriately performed? |  |  |  | N/A |
|  | 2.2. Are the groups comparable at baseline? |  |  |  | N/A |
|  | 2.3. Are there complete outcome data? |  |  |  | N/A |
|  | 2.4. Are outcome assessors blinded to the intervention provided? |  |  |  | N/A |
|  | 2.5 Did the participants adhere to the assigned intervention? |  |  |  | N/A |
| 3. Quantitative non- randomized | 3.1. Are the participants representative of the target population? | X |  |  |  |
|  | 3.2. Are measurements appropriate regarding both the outcome and intervention (or exposure)? | X |  |  |  |
|  | 3.3. Are there complete outcome data? | X |  |  |  |
|  | 3.4. Are the confounders accounted for in the design and analysis? |  | X |  |  |
|  | 3.5. During the study period, is the intervention administered (or exposure occurred) as intended? | X |  |  |  |
| 4. Quantitative descriptive | 4.1. Is the sampling strategy relevant to address the research question? |  |  |  | N/A |
|  | 4.2. Is the sample representative of the target population? |  |  |  | N/A |
|  | 4.3. Are the measurements appropriate? |  |  |  | N/A |
|  | 4.4. Is the risk of nonresponse bias low? |  |  |  | N/A |
|  | 4.5. Is the statistical analysis appropriate to answer the research question? |  |  |  | N/A |
| 5. Mixed methods | 5.1. Is there an adequate rationale for using a mixed methods design to address the research question? | X |  |  |  |
|  | 5.2. Are the different components of the study effectively integrated to answer the research question? | X |  |  |  |
|  | 5.3. Are the outputs of the integration of qualitative and quantitative components adequately interpreted? | X |  |  |  |
|  | 5.4. Are divergences and inconsistencies between quantitative and qualitative results adequately addressed? |  |  | X |  |
|  | 5.5. Do the different components of the study adhere to the quality criteria of each tradition of the methods involved? | X |  |  |  |

***Table 11.*** *The following tables show the quality assessment for mixed method study with the Mixed Methods Appraisal Tool (MMAT), version 2018*

| **Pani et al. (2017)** | **Methodological quality criteria** | **Responses** | | | |
| --- | --- | --- | --- | --- | --- |
|  |  | Yes | No | Can’t tell | Comments |
| Screening questions (for all types) | S1. Are there clear research questions? | X |  |  |  |
|  | S2. Do the collected data allow to address the research questions? | X |  |  |  |
|  | Further appraisal may not be feasible or appropriate when the answer is ‘No’ or ‘Can’t tell’ to one or both screening questions. | | | | |
| 1. Qualitative | 1.1. Is the qualitative approach appropriate to answer the research question? |  |  | X |  |
|  | 1.2. Are the qualitative data collection methods adequate to address the research question? |  |  | X |  |
|  | 1.3. Are the findings adequately derived from the data? | X |  |  |  |
|  | 1.4. Is the interpretation of results sufficiently substantiated by data? | X |  |  |  |
|  | 1.5. Is there coherence between qualitative data sources, collection, analysis and interpretation? |  |  | X |  |
| 2. Quantitative randomized controlled trials | 2.1. Is randomization appropriately performed? |  |  |  | N/A |
|  | 2.2. Are the groups comparable at baseline? |  |  |  | N/A |
|  | 2.3. Are there complete outcome data? |  |  |  | N/A |
|  | 2.4. Are outcome assessors blinded to the intervention provided? |  |  |  | N/A |
|  | 2.5 Did the participants adhere to the assigned intervention? |  |  |  | N/A |
| 3. Quantitative non- randomized | 3.1. Are the participants representative of the target population? |  |  |  | N/A |
|  | 3.2. Are measurements appropriate regarding both the outcome and intervention (or exposure)? |  |  |  | N/A |
|  | 3.3. Are there complete outcome data? |  |  |  | N/A |
|  | 3.4. Are the confounders accounted for in the design and analysis? |  |  |  | N/A |
|  | 3.5. During the study period, is the intervention administered (or exposure occurred) as intended? |  |  |  | N/A |
| 4. Quantitative descriptive | 4.1. Is the sampling strategy relevant to address the research question? |  | X |  |  |
|  | 4.2. Is the sample representative of the target population? |  |  | X |  |
|  | 4.3. Are the measurements appropriate? |  |  | X |  |
|  | 4.4. Is the risk of nonresponse bias low? |  |  | X |  |
|  | 4.5. Is the statistical analysis appropriate to answer the research question? |  | X |  |  |
| 5. Mixed methods | 5.1. Is there an adequate rationale for using a mixed methods design to address the research question? |  |  | X |  |
|  | 5.2. Are the different components of the study effectively integrated to answer the research question? | X |  |  |  |
|  | 5.3. Are the outputs of the integration of qualitative and quantitative components adequately interpreted? | X |  |  |  |
|  | 5.4. Are divergences and inconsistencies between quantitative and qualitative results adequately addressed? |  |  | X |  |
|  | 5.5. Do the different components of the study adhere to the quality criteria of each tradition of the methods involved? |  |  | X |  |

***Table 12.*** *The following tables show the quality assessment for mixed method study with the Mixed Methods Appraisal Tool (MMAT), version 2018*

| **Shewchuk et al (2021)** | **Methodological quality criteria** | **Responses** | | | |
| --- | --- | --- | --- | --- | --- |
|  |  | Yes | No | Can’t tell | Comments |
| Screening questions (for all types) | S1. Are there clear research questions? | X |  |  |  |
|  | S2. Do the collected data allow to address the research questions? | X |  |  |  |
|  | Further appraisal may not be feasible or appropriate when the answer is ‘No’ or ‘Can’t tell’ to one or both screening questions. | | | | |
| 1. Qualitative | 1.1. Is the qualitative approach appropriate to answer the research question? | X |  |  |  |
|  | 1.2. Are the qualitative data collection methods adequate to address the research question? |  |  | X |  |
|  | 1.3. Are the findings adequately derived from the data? | X |  |  |  |
|  | 1.4. Is the interpretation of results sufficiently substantiated by data? | X |  |  |  |
|  | 1.5. Is there coherence between qualitative data sources, collection, analysis and interpretation? | X |  |  |  |
| 2. Quantitative randomized controlled trials | 2.1. Is randomization appropriately performed? |  |  |  | N/A |
|  | 2.2. Are the groups comparable at baseline? |  |  |  | N/A |
|  | 2.3. Are there complete outcome data? |  |  |  | N/A |
|  | 2.4. Are outcome assessors blinded to the intervention provided? |  |  |  | N/A |
|  | 2.5 Did the participants adhere to the assigned intervention? |  |  |  | N/A |
| 3. Quantitative non- randomized | 3.1. Are the participants representative of the target population? |  |  |  | N/A |
|  | 3.2. Are measurements appropriate regarding both the outcome and intervention (or exposure)? |  |  |  | N/A |
|  | 3.3. Are there complete outcome data? |  |  |  | N/A |
|  | 3.4. Are the confounders accounted for in the design and analysis? |  |  |  | N/A |
|  | 3.5. During the study period, is the intervention administered (or exposure occurred) as intended? |  |  |  | N/A |
| 4. Quantitative descriptive | 4.1. Is the sampling strategy relevant to address the research question? |  |  | X |  |
|  | 4.2. Is the sample representative of the target population? |  |  | X |  |
|  | 4.3. Are the measurements appropriate? | X |  |  |  |
|  | 4.4. Is the risk of nonresponse bias low? |  |  | X |  |
|  | 4.5. Is the statistical analysis appropriate to answer the research question? |  |  | X |  |
| 5. Mixed methods | 5.1. Is there an adequate rationale for using a mixed methods design to address the research question? | X |  |  |  |
|  | 5.2. Are the different components of the study effectively integrated to answer the research question? | X |  |  |  |
|  | 5.3. Are the outputs of the integration of qualitative and quantitative components adequately interpreted? |  |  | X |  |
|  | 5.4. Are divergences and inconsistencies between quantitative and qualitative results adequately addressed? |  |  | X |  |
|  | 5.5. Do the different components of the study adhere to the quality criteria of each tradition of the methods involved? |  |  | X |  |
